# Environmental Enrichment Therapy for Autism: Outcomes with Increased Access

**DOI:** 10.1155/2016/2734915

**Published:** 2016-09-18

**Authors:** Eyal Aronoff, Robert Hillyer, Michael Leon

**Affiliations:** ^1^Mendability, LLC, 915 South 500 East, American Fork, UT 84003, USA; ^2^Center for Autism Research and Translation, Center for the Neurobiology of Learning and Memory, Department of Neurobiology and Behavior, 2205 McGaugh Hall, The University of California Irvine, Irvine, CA 92697-4550, USA

## Abstract

We have previously shown in two randomized clinical trials that environmental enrichment is capable of ameliorating symptoms of autism spectrum disorder (ASD), and in the present study, we determined whether this therapy could be effective under real-world circumstances. 1,002 children were given daily Sensory Enrichment Therapy, by their parents, using personalized therapy instructions given over the Internet. Parents were asked to assess the symptoms of their child every 2 weeks for up to 7 months. An intention-to-treat analysis showed significant overall gains for a wide range of symptoms in these children, including learning, memory, anxiety, attention span, motor skills, eating, sleeping, sensory processing, self-awareness, communication, social skills, and mood/autism behaviors. The children of compliant caregivers were more likely to experience a significant improvement in their symptoms. The treatment was effective across a wide age range and there was equal progress reported for males and females, for USA and international subjects, for those who paid and those who did not pay for the therapy, and for individuals at all levels of initial symptom severity. Environmental enrichment, delivered via an online system, therefore appears to be an effective, low-cost means of treating the symptoms of ASD.

## 1. Introduction

Autism spectrum disorder (ASD) is a heterogeneous neurodevelopmental condition, presenting in early childhood in 1 of 45 children in the USA [1], and it appears to arise from a complex interaction between genetic and environmental factors [2–4]. Social interaction and communication skills are impaired in this disorder, and individuals with ASD also have unusual repetitive behaviors and/or narrow interests. The condition can persist for life, with major implications for the individual, the family, and the healthcare system [5]. There are currently limited medical treatments for individuals with ASD [6], and while there are several behavioral therapies available for treatment, these programs are inaccessible to many [7, 8], are often costly [5, 9], are typically less effective as patients age [10], are not reliably effective [11], and may address a narrow range of symptoms [12–15]. A treatment that successfully addresses the limitations of current therapies therefore would be of great value.

How much can the environment affect the expression of ASD symptoms? After reviewing the animal literature regarding the substantial benefits of environmental enrichment for animal models of autism, Reynolds et al. [16] noted that the key aspects of environmental enrichment appear to include novel and diverse sensorimotor experiences. They went on to propose that environmental enrichment might be a useful means of treating children with autism, presumably by suppressing the expression of ASD symptoms through neural compensatory mechanisms that would be evoked by the environmental stimulation. In other words, the gene x environment interaction that produced the expression of autism symptoms may be shifted by changing the environmental input to the children with ASD and thereby reducing the expression of those symptoms.

To test that hypothesis, we conducted a randomized clinical trial in which environmental enrichment was given to 6–13-year-old children with classic autism for 6 months by their parents [17]. The therapy included about three-dozen novel sensory exercises that were given to the children in the morning and evening. The children were assessed at baseline and after 6 months by the same psychologists who were unaware of the group assignment of the children. We found clinically significant improvements in autism symptom severity in 42% of the children in the enriched group, as revealed by the Childhood Autism Rating Scale, while only 7% of the standard-care controls experienced such an improvement. We also reported improved cognition following environmental enrichment, with enriched children scoring 11.3 points higher than controls on the objective Leiter International Performance Scale (Leiter-R) after 6 months of therapy.

In a second randomized clinical trial, environmental enrichment was used to treat 3–6-year-old children with classic autism, and their assessments were also completed by experienced psychologists who were blind to group assignment. Woo et al. [18] again found significant improvements in the cognitive scores of enriched children over 6 months using the Leiter-R, with enriched children gaining 8.42 IQ points, while the standard-care group gained 1.53 points, a statistically significant difference. A significant improvement for the enriched children was also found in receptive language, using the Reynell Developmental Language Scales, another objective test of symptom improvement. Enriched children gained 7.42 points on the receptive language scale, whereas the standard-care group had an average increase of 3.63 points on that assessment. Improvements for the enriched children also outpaced that of controls in the reduction of abnormal sensory responsiveness, as assessed by their parents with the Short Sensory Profile [19]. Enriched children gained 11.36 points on that survey, whereas the standard-care children improved by 2.85 points. Finally, we found that 21% of the children who were initially classified as having autism with the objective Autism Diagnostic Observation Schedule fell below the autism classification cutoff using that same test after six months of environmental enrichment. None of the children in the standard-care control group improved to that extent. In both of these studies, standard care included various combinations of speech therapy, occupational therapy, Applied Behavioral Analysis, social skills therapy, adapted physical education, and physical therapy.

We then wanted to determine whether this therapy could be provided to a large number of individuals with autism via a telehealth system under real-world conditions. Indeed, several forms of behavioral therapy have been made available to relatively small numbers of parents via the Internet with encouraging outcomes. Parental instruction for autism behavioral therapies such as Pivotal Response Training, Applied Behavior Analysis, and the Early Start Denver Protocol have been made available over the Internet and these at-home therapies have shown significant improvements in parental confidence and parental treatment fidelity, as well as improvements in spontaneous imitation skills, communication skills, problem behaviors, and anxiety in children with ASD [20–28].

Given the efficacy of environmental enrichment for treatment of ASD symptom in our randomized clinical trials and given its potential for increasing access to treatment for children with ASD, the enrichment therapy has been adapted for real-world circumstances and made available on the Internet by Mendability, LLC, as a paid online service, which has been accredited for the provision of behavioral healthcare by the Joint Commission on Accreditation of Healthcare Organizations. We set out to determine the efficacy of environmental enrichment as provided via this telehealth service. Unlike the therapy used in the two clinical trials, the therapeutic exercises from this system were individualized, based upon the specific challenges, age, abilities, and progress of the children. In addition, while both of the clinical trials included only children 3–12 years old with classic autism, this study extended both the range of ages of the subjects who received the therapy and the range of ASD severity of those treated by including self-selected individuals across the entire autism spectrum.

## 2. Materials and Methods

### 2.1. Intervention

All research activities adhered to the Health Insurance Portability and Accountability Act and were in compliance with its privacy, security, and electronic transaction guidelines. The data set used in the study was stripped of personal information prior to analysis.

The study was a nonconcurrent single-subject, multiple-baseline design initiated by a retrospective review of the behavioral assessments of parents regarding their children at the start of their treatment and over the course of their treatment. The individuals who received environmental enrichment were given instructions for daily exposure to multiple sensorimotor exercises via customized worksheets that were generated by the online software after completion of an extensive questionnaire. As the parents were delivering the stimulation, there was also an increase in social interactions for the enriched children. Licensed, experienced occupational therapists reviewed the worksheets to ensure that the computer-generated exercises were within the capability of each subject. These therapists made any adjustments to the therapy if needed, but such interventions were rare. The occupational therapists were also available for consultations with the parents via email, phone, and video over the course of the therapeutic intervention

Environmental enrichment, in the form of Sensory Enrichment Therapy, pairs different types of sensory and motor exercises on a daily basis. Varied textures, such as plastic turf doormats, aluminum foil, sponges, artificial flowers, adhesive tape, and bubble wrap, were used to stimulate the sense of touch. For object manipulation, there were beads to sort and arrange, discs to insert or pull, and rice or toothpicks to insert into foam or Play-Doh, which was also used to squeeze and shape. Thermal stimulation came from different temperatures of water, spoons, or mugs. Visual stimulation came in the form of fine art, photos, and other images. Auditory stimulation came in the form of classical music or sound makers. Proprioceptive and vestibular stimulation came in the form of various exercises requiring walking or ascending and descending stairs while carrying an object overhead. Balance skills were elicited on a raised or angled beam, and different movements were performed in place with a blindfold. Pleasant scents provided olfactory stimulation. A partial list of the exercises available to the children in this study can be found in previous reports [17, 18]. The online system selected exercises from a database of more than 400 different sensory exercises, which allowed a new individualized therapy worksheet to be developed for each 2-week period.

The therapeutic exercises were administered once or twice a day, with each session lasting 10–15 minutes. In addition, there were 4–6 daily pairings of olfactory stimuli and gentle tactile stimuli for 30–60 seconds. Every 2 weeks, after the detailed parental assessments of their child's symptoms were completed, the expert system software assigned new exercises that were delivered in a new worksheet for the next 2-week period. The enrichment therapy was added to any other therapies in which the child was engaged.

### 2.2. Assessments

Parents were initially asked to complete an online assessment with 301 potential questions that probed the behavioral symptoms associated with autism (a list of which can be found in the Appendix). Care was given to avoid questions that could be extracted from higher-level questions. For example, if the parent reported that the child had good reading comprehension, the system did not ask if she/he knew the letters of the alphabet. Furthermore, parents were presented only age-appropriate questions. For example, if the parent reported that the child was 3 years old, a question about reading comprehension would not be presented. The parents were asked to assess each aspect of their behavior with these descriptors: 0 = could not be worse, 1 = severe problem, 2 = big problem, 3 = bit of a problem, 4 = maybe a problem, and 5 = not a problem. A progress bar was displayed for each question to help the parent set the level of improvement on the scale.

In the initial assessment, the system presented an average of 280.46 (SD = 22.43, CI = 279.07–281.85) questions. Whenever the parent rated a question as “not a problem,” (mean = 153 or 55% of the questions, SD = 48.81, CI = 150–156) or when the parent was not able to generate an answer to a question for various reasons (mean = 8 questions or 3% of the questions, SD = 16, CI = 7–9), those questions were then omitted from the subsequent questionnaires.

### 2.3. Participation

1,002 subjects were recruited to initiate the treatment, using Google ads, TEDx ads, Facebook ads, and email messages. They had a mean age of 7.37 years (SD = 3.83, CI = 7.14–7.61) and ranged in age between 1 and 18 years. There were 796 males and 206 females, 752 of whom were from the USA, 239 were international residents, and 11 were of unknown geographic location. There were 835 children whose parents paid for the therapy and 167 children who received the therapy at no charge. There were 559 parents who indicated that their child had received a diagnosis of autism, 41 children who had a diagnosis of Asperger's syndrome, and 30 who had probable autism. In addition, 31 children were regarded as having pervasive developmental disorder, 18 were regarded as having ADHD, 10 were described as having global developmental delay, 42 were described as having other disorders, and there were 271 children whose parents did not provide a formal diagnosis. However, rather than focusing on their presumptive diagnoses, we focused on improvement in the symptoms that were revealed by the answers to the assessment questions.

### 2.4. Calculating Composite Score

The parents completed these questionnaires a mean of 1.75 times each month. The mean number of questions to which the parents responded over the course of their participation was 119 questions at each assessment (SD = 43.54, CI = 116–122). The final scores were recorded for each individual whenever they stopped their participation and the change in symptom severity was then calculated for each subject for all answered questions in an intention-to-treat analysis. The range of scores for the individual behavioral components was 0 to 5. The mean of all answered questions was then calculated for a composite score that characterized the mean change in symptom severity for each child, as assessed by their parent. Questions that were not age-appropriate or were initially answered as “not a problem” or “cannot measure” were not subsequently presented and were therefore not included in the calculations. If no assessment was taken in any month, we interpolated the results by time-weighted averaging between the last assessment and the next closest assessment. In addition, we calculated the change in assessments for specific categories of symptoms: anxiety, attention span, communication, eating, learning, memory, mood/behavior, motor skills, self-awareness, sensory processing, sleep, and social skills. We further clustered those categories into basic skills, complex skills, and personality traits for analysis. We used paired* t*-tests for two sample means to compare symptom severity before and after Sensory Enrichment Therapy, and we calculated* R* ^2^ for evaluating correlations.

## 3. Results and Discussion

174 participants answered the questionnaire for 1 month, 144 for two months, 81 for 3 months, 65 for 4 months, 79 for 5 months, 59 for 6 months, and 400 for more than 6 months. In all, we collected more than 650,000 answers to the questions that are shown in the Appendix, along with the proportion of subjects who indicated a problem in that area, the mean initial score, and the mean change in that score at the final assessment.

**Figure 1:**
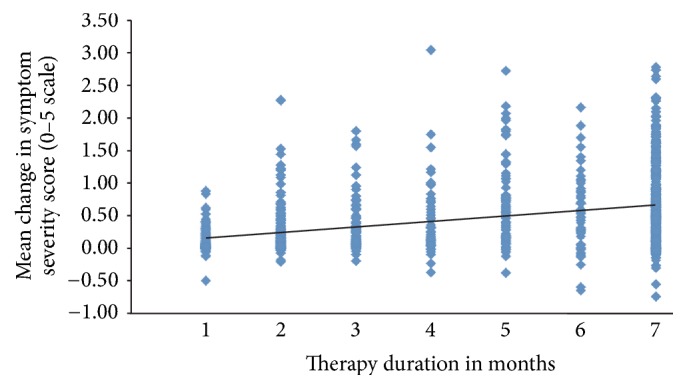
Mean change in symptom severity score as a function of therapy duration in months (*R* ^2^ = 0.14). Symptom severity score was the change on a 0–5 scale for all answered questions for each subject.

Figure 1 shows the correlation of the time spent in environmental enrichment therapy in months, relative to the mean difference in the composite score of the participants from the initiation of the therapy to their final assessment:* R* ^2^ = 0.14 (*p* < 0.05). The mean initial symptom severity score for all subjects was 2.48 (SD = 0.60, CI = 2.44–2.51) and the mean final score for all subjects improved to 2.93 (SD = 0.78, CI = 2.88–2.98; df = 1,001,* t* = −26.58, *p* < 0.00001). The effect size should be considered to be large (Cohen's* d* = −1.68).

**Figure 2:**
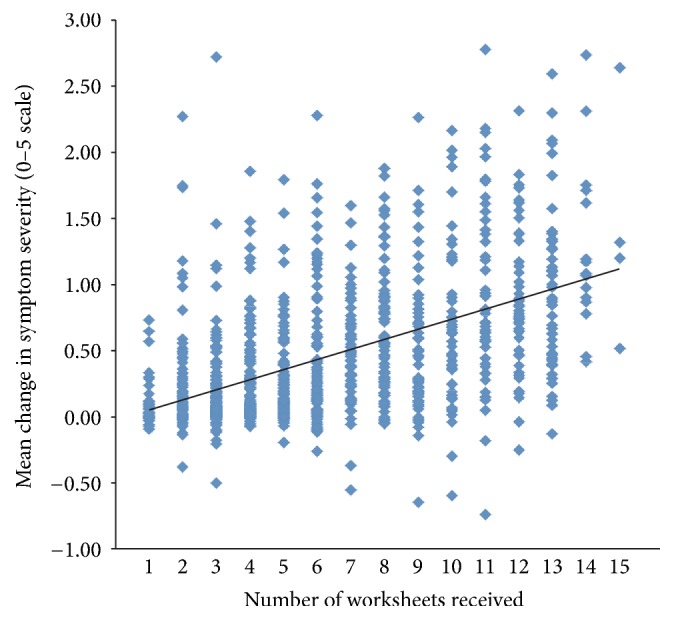
Mean change in symptom severity, as determined by all answered questions on a 0–5 scale from the initial to the final assessment as a function of the number of worksheets received, which served as a reflection of parental engagement with the therapy (*R* ^2^ = 0.26).

**Figure 3:**
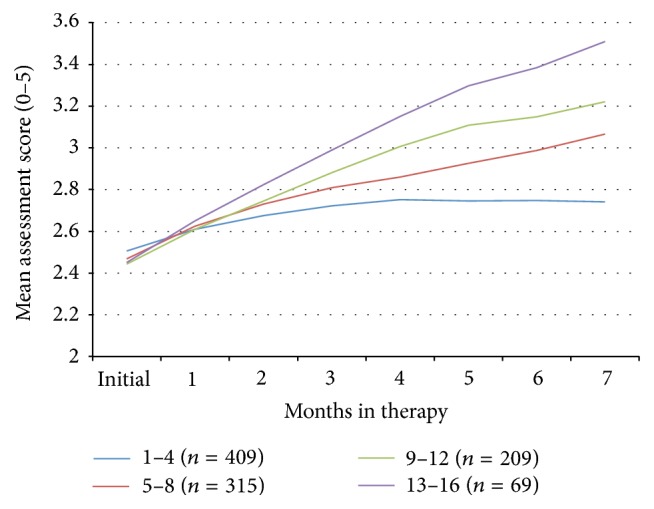
Mean composite assessment score (scale 0–5) over time for subjects whose parents downloaded different numbers of exercise worksheets.

The change in symptom severity as a function of parental engagement with the therapy, as determined by the number of sensory exercise worksheets that the parents downloaded, is shown in Figures 2 and 3. The more assumed parental engagement, the better outcome for the children (*R* ^2^ = 0.26, *p* < 0.05). Indeed, only 5.93% (2,036 out of 35,055) of questions answered by the 295 parents who downloaded 1–3 worksheets had experienced an improvement of at least 1 point on the symptom severity scale, while 46.09% (11,467 out of 24,852) of questions answered by the 217 parents who downloaded at least 10 worksheets had such an improvement. Even though the children of parents who downloaded 1–3 exercise sheets had a significant improvement in their progress on their composite scores of symptom severity (mean = 0.18, SD = 0.34, CI = 0.14–0.22,* t* = −9.00, df = 294, *p* < 0.00001), those subjects whose parents downloaded 10 or more exercise sheets had a mean improvement of 0.90 (SD = 0.63, CI = 0.81–0.98,* t* = −20.95, df = 216, *p* < 0.00001). When the symptom improvement for both of these groups was compared directly with an unpaired* t*-test with unequal variances, the children of the compliant parents had a significantly better outcome than the children of the noncompliant parents (*t* = −15.19, df = 309, *p* < 0.00001).

To understand the impact of the passage of time on symptom severity, we looked at the progress of 93 subjects whose parents had completed the assessments for 6 or more months but only downloaded 1–3 worksheets. These data differ from the analysis above in that we only looked at those children who had the therapy for at least 6 months, whereas the above comparison included the outcomes of all the children in these groups, regardless of the time that they remained in the study. This noncompliant group had a mean age of 7.77 (SD = 3.93, CI = 6.96–8.58) and a mean initial severity of 2.53 (SD = 0.65, CI = 2.40–2.67) and the percent of questions that were initially marked as “not a problem” was 54% (SD = 17%, CI = 51%–58%), with mean composite symptom improvement of 0.24 (SD = 0.35, CI = 0.17–0.31,* t* = −6.72, df = 92, *p* < 0.00001). Due to the low compliance levels, this group likely reflects the improvement of symptoms over time without significant Sensory Enrichment Therapy, compared to the 182 subjects whose parents completed assessments for 6 or more months and downloaded 10 or more worksheets. That group had similar mean age: 7.46 (SD = 3.87, CI = 6.89–8.02), similar mean initial severity: 2.45 (SD = 0.57, CI = 2.36–2.53), and similar percent of initial questions that were marked as “not a problem”: 55% (SD = 16%, CI = 52%–57%), but they had a mean composite symptom improvement of 0.91 (SD = 0.63, CI = 0.82–1.00,* t* = −19.40, df = 181, *p* < 0.00001). A direct comparison of these groups showed that the compliant parents had children who experienced much larger symptom improvements than the noncompliant parents even when time is kept constant (*t* = −11.28, df = 272, *p* < 0.00001).

**Figure 4:**
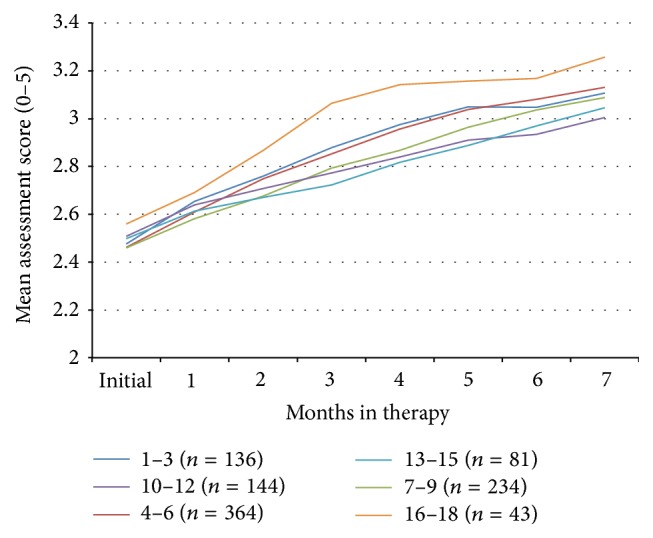
Mean composite assessment score (scale 0–5) over time for subjects of different ages.

**Figure 5:**
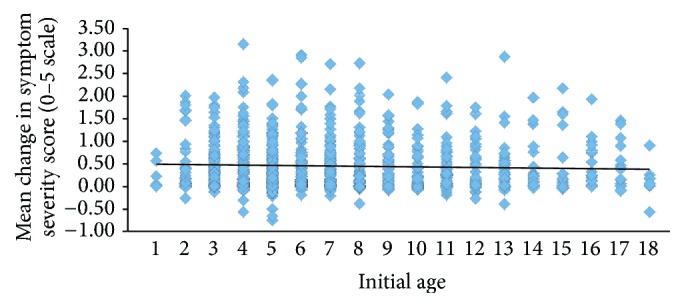
Mean change in symptom severity score as a function of initial age (*R* ^2^ = 0.002). Symptom severity score was the change on a 0–5 scale for all answered questions for each subject.

**Figure 6:**
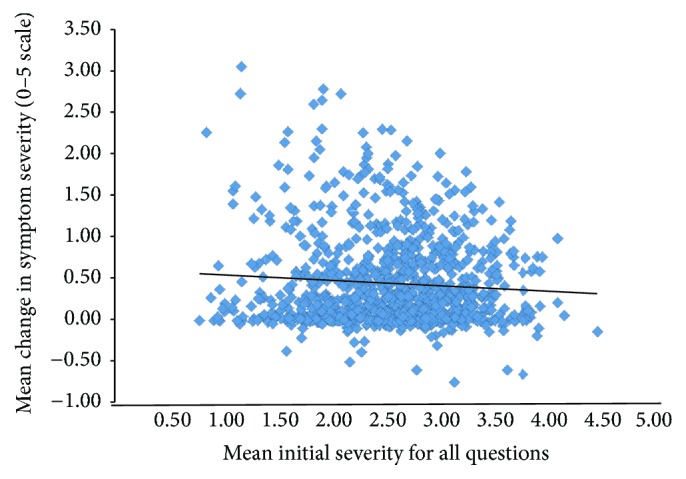
Mean change in symptom severity, determined by all answered questions on a 0–5 scale as a function of initial symptom severity (*R* ^2^ = 0.005).

**Figure 7:**
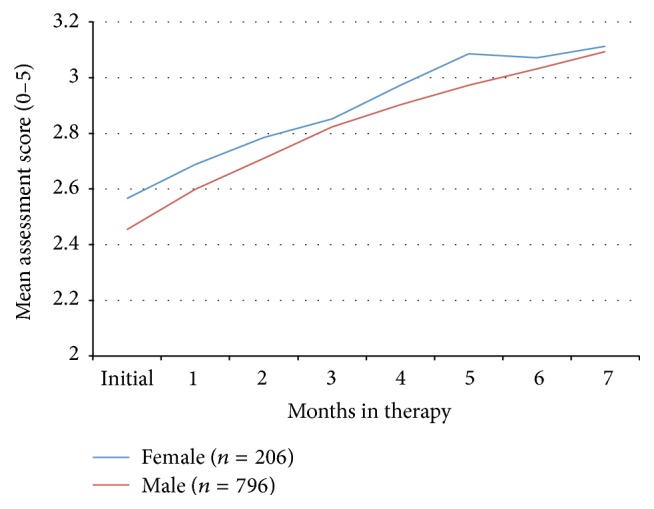
Mean composite assessment score (scale 0–5) over time for male and female subjects.

The change in symptom severity as a function of initial age is shown in Figures 4 and 5 and there was no statistically significant difference between these factors. Initial symptom severity also did not affect the eventual outcomes (Figure 6). Subjects with a composite symptom severity score < 3 (mean change = 0.47, SD = 0.56, CI = 0.43–0.51,* t* = −23.56, df = 802, *p* < 0.00001) and subjects with a composite symptom severity score > 3 (mean change = 0.40, SD = 0.44, CI = 0.33–0.46,* t* = −12.75, df = 198, *p* < 0.00001) had similar significant improvements in their symptoms. Both American subjects (mean = 0.45, SD = 0.55, CI = 0.41–0.49,* t* = −22.53, df = 751, *p* < 0.00001) and international subjects (mean = 0.45, SD = 0.51, CI = 0.38–0.51,* t* = −13.54, df = 238, *p* < 0.00001), along with both males (mean = 0.46, SD = 0.55, CI = 0.42–0.50,* t* = −23.74, df = 795, *p* < 0.00001) and females (mean = 0.43, SD = 0.52, CI = 0.36–0.50,* t* = −11.86, df = 205, *p* < 0.00001), experienced similar improvements in their symptoms (Figure 7).

Those symptoms that we regarded as basic skills improved by a mean of 0.49 (SD = 0.55, CI = 0.46–0.53,* t* = −28.19, df = 1,001, *p* < 0.00001), complex skills by 0.42 (SD = 0.55, CI = 0.38–0.45,* t* = −23.99, df = 1,000, *p* < 0.00001), and personality traits by 0.35 (SD = 0.59, CI = 0.32–0.39,* t* = −18.87, df = 990, *p* < 0.00001).

We found statistically significant improvements for all symptom categories: anxiety (mean score improvement = 0.43, SD = 0.67, CI = 0.38–0.47,* t* = −18.79, df = 864), attention span (mean = 0.42, SD = 0.68, CI = 0.38–0.46,* t* = −19.12, df = 955), communication (mean = 0.51, SD = 0.65, CI = 0.47–0.55,* t* = −23.55, df = 920), eating (mean = 0.48, SD = 0.68, CI = 0.43–0.52,* t* = −19.66, df = 782), learning (mean = 0.47, SD = 0.63, CI = 0.43–0.51,* t* = −22.77, df = 935), memory (mean = 0.41, SD = 0.69, CI = 0.35–0.47,* t* = −14.38, df = 576), mood/behavior (mean = 0.40, SD = 0.61, CI = 0.36–0.43,* t* = −20.28, df = 970), motor skills (mean = 0.45, SD = 0.58, CI = 0.41–0.49,* t* = −22.82, df = 875), self-awareness (mean = 0.50, SD = 0.72, CI = 0.45–0.55,* t* = −20.75, df = 882), sensory processing (mean = 0.49, SD = 0.62, CI = 0.45–0.53,* t* = −24.24, df = 954), sleep (mean = 0.47, SD = 0.67, CI = 0.42–0.53,* t* = −18.19, df = 654), and social skills (mean = 0.42, SD = 0.61, CI = 0.38–0.45,* t* = −21.40, df = 972). All* p*'s < 0.00001. The* n* differs in these comparisons because not all subjects had problems in all areas of concern.

There was no significant difference in the initial characteristics between the children whose parents paid for the treatment (*n* = 835, mean age = 7.21 years, SD = 3.76, CI = 6.96–7.47), initial composite symptom severity (mean = 2.48, SD = 0.59, CI = 2.44–2.52) and percent of questions marked as “not a problem” (mean = 55%, SD = 17%, CI = 53%–56%), and those who did not pay for the treatment (*n* = 167, mean age = 8.17 years, SD = 4.08, CI = 7.54–8.79), mean initial severity (mean = 2.47, SD = 0.65, CI = 2.37–2.57) and percent of questions marked as “not a problem” (mean = 54%, SD = 17%, CI = 52%–57%). Similarly, there was no significant difference in improvement between the paying group and the nonpaying group. The mean change in composite scores was 0.45 (*n* = 835, SD = 0.55, CI = 0.41–0.49,* t* = −23.79, df = 834, *p* < 0.00001) for those who paid and 0.47 (*n* = 167, SD = 0.51, CI = 0.39–0.55,* t* = −11.90, df = 166, *p* < 0.00001) for those who did not pay for the treatment.

**Figure 8:**
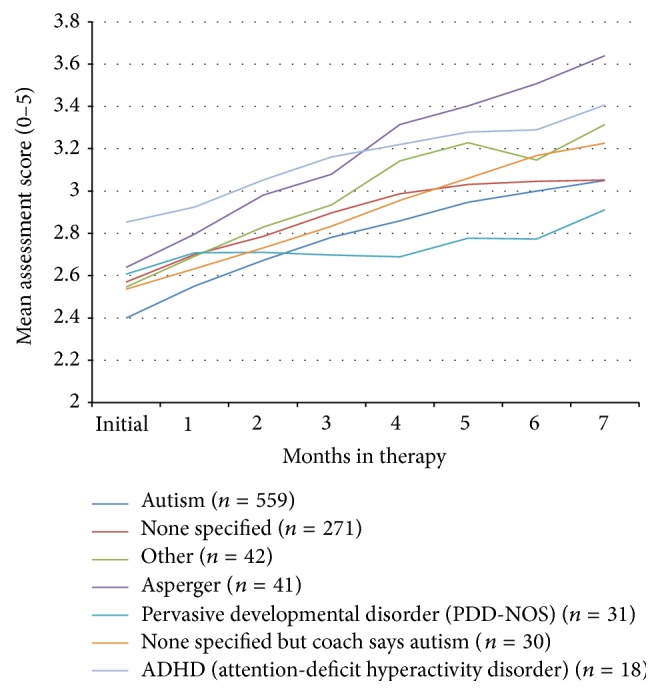
Mean composite assessment score (scale 0–5) over time for subjects with different reported diagnoses.

The progress of those subjects with different reported diagnoses is shown in Figure 8. Those reported to have ADHD had a mean improvement in their symptoms of 0.33 (SD = 0.41, CI = 0.13–0.54,* t* = −3.46, df = 17, *p* < 0.003). Those described as having Asperger's syndrome had a significant improvement in their symptoms (mean improvement = 0.54, SD = 0.61, CI = 0.34–0.73,* t* = −5.63, df = 40, *p* < 0.00001), those with ASD diagnoses improved by 0.47 (SD = 0.58, CI = 0.43–0.52,* t* = −19.25, df = 558, *p* < 0.00001), and those who were regarded as having ASD by the occupational therapists (coach) improved by 0.70 (SD = 0.46, CI = 0.53–0.87,* t* = −8.40, df = 29, *p* < 0.0001). Those with global developmental delay also benefitted from the treatment, with a mean composite severity score improvement of 0.56 (SD = 0.77, CI = 0.01–1.11,* t* = −2.31, df = 9, *p* < 0.05). Those individuals who were described as having PDD-NOS improved by 0.33 (SD = 0.36, CI = 0.20–0.46,* t* = −5.18, df = 30, *p* < 0.0001). Subjects without a reported diagnostic category improved by 0.39 (SD = 0.46, CI = 0.33–0.44,* t* = −13.69, df = 270, *p* < 0.00001) and those who were regarded as being in a variety of diagnostic categories improved by 0.47 (SD = 0.44, CI = 0.33–0.60,* t* = −6.95, df = 41, *p* < 0.00001).

### 3.1. Treatment Outcomes

Environmental enrichment in the form of Sensory Enrichment Therapy, provided through an online portal, appears to be effective in supporting a broad range of symptomatic improvements in individuals with ASD over a wide range of ages and symptom severity, as well as across geographic locations, and for both genders. There was also a significant association between treatment compliance and therapeutic effectiveness.

The online treatment effects even appear to be stronger than what was observed in the randomized clinical trials, where the treatment effects based on Cohen's* d* for the standardized mean difference for the within-subject change on the Leiter, Short Sensory Profile, and Reynell Receptive Language assessments were 0.54, 0.51, and 0.45, respectively [18], outcomes that should be regarded as producing medium/large effects. The magnitude of the effect for improvement in composite scores using the online system for all participants was 1.68, a large effect. The improvement in effect size over the randomized clinical trials, all things being equal, raises the possibility that the personalized sensorimotor exercises used in the online system may have been superior to the standardized exercises that were used in the randomized clinical trials. The advice and guidance of occupational therapists also may have contributed to the efficacy of the online system. Parents may also have been hopeful for a positive outcome for their child and that hope may have been reflected in their assessments. It will be important, therefore, to conduct a further study to test children treated with the online system with objective, validated assessments.

The outcomes in this study compare well with other parent-mediated therapies, particularly for those therapies geared for children and adolescents [29–34]. Moreover, environmental enrichment appears to benefit both core ASD symptoms and symptoms that are typically comorbid with autism [35, 36]. Indeed, 92% of children with ASD have at least two cooccurring mental health problems [37].

It is also of interest that children who were at different levels of symptom severity at the initiation of the therapy were able to benefit equally from this therapeutic approach. Similarly, both males and females benefitted from this approach to the same extent. Perhaps the most compelling finding is that older individuals benefitted to the same extent as younger subjects. Given that standard-care interventions are typically effective principally for young children [10, 38, 39], it is encouraging to have a therapy that is effective over a wide range of ages.

### 3.2. Sensory Impairment and ASD

We have again shown that enhanced sensorimotor experiences appear to ameliorate ASD symptoms. Conversely, it also appears that a degradation of sensory experiences may increase the risk of autism. For example, congenitally blind children have a 42% elevated probability of having an ASD diagnosis [40]. Even children with less serious ophthalmic problems have a 19% elevated risk of ASD [41]. Indeed, 69% of children with ASD were reported to have abnormal visual acuity [42]. Individuals with autism also have deficits in visual motion processing, as assessed by fMRI responses, which accompany deficits in both primary visual cortex and extrastriate cortex [43]. Similarly, visually evoked electrophysiological potentials reveal neural responses early in the visual pathway that are compromised in individuals with ASD [44].

It is also the case that degradation of auditory stimulation is associated with an increased risk of ASD. Up to 7% of deaf children are diagnosed with ASD [45, 46] and 10% of individuals with ASD were reported to have hearing problems [47].

Möbius syndrome typically involves both hearing loss and visual difficulties, and 45% of these children are diagnosed with ASD [48, 49]. The congenital oculo-auriculo-vertebral spectrum disorder also involves loss of vision and audition and 42% of those individuals are given an ASD diagnosis [50]. Furthermore, 68% of children with CHARGE syndrome have ASD diagnoses, which involves an even greater multisensory loss (hearing, olfaction, and vision) [51, 52]. Sensory loss therefore is associated with an increased risk of the expression of ASD symptoms and greater sensory loss is associated with a higher ASD risk.

Individuals with ASD also have problems integrating multisensory information into a single percept [53–56]. Using diffusion tensor imaging fiber tractography, Chang et al. [57] evaluated the structural connectivity of white matter tracts in individuals with ASD and they found that they had decreased connectivity relative to controls in parietooccipital tracts involved in sensory perception and multisensory integration.

Since the loss of sensory stimulation due to neural anomalies or damage to sensory systems is associated with the increased expression of ASD symptoms, it seems possible that environmental restriction of sensory stimuli would have a similar effect. Indeed, a significant proportion of children who were raised in orphanages with very little sensory or social stimulation develop what has been called postinstitutional autistic syndrome [58]. Such children display symptoms similar to children with ASD: they have stereotypic behaviors, an inability to identify human emotions, disordered social communication, abnormal language, poor cognition, abnormal executive function, altered theory of mind, poor sensory integration, poor motor behavior, and abnormal attachment responses [58–63].

These children also share some of the same neurological abnormalities as children with autism. For example, both groups have depressed activity in their orbitofrontal cortex/amygdala circuit, areas associated with social cognition and emotion [64–67]. Children raised in orphanages and children with ASD also have diminished white-matter connectivity in the uncinate fasciculus [68–71], which is a major pathway for communication between the amygdala and orbitofrontal cortex. Both socially/sensory deprived children and children with ASD do not have the right-hemisphere specialization for their neurophysiological response to human faces [72–76]. Finally, neither children with ASD nor institutionalized children have the normal increase in ventral striatum activity as they anticipate a reward [77–81].

The importance of sensory/social stimulation was underlined in the deprived children when they were placed in foster homes or were given environmental enrichment in their orphanage. In these new circumstances, many of their symptoms were greatly ameliorated and their cognitive abilities improved significantly [82–84]. Moreover, the quality of the foster care correlates with better improvements in their outcomes, as does their early transfer into foster care [59, 85, 86]. Sensory deprivation therefore appears to be associated with an increased risk of expressing ASD symptoms and sensory enrichment seems to be able to ameliorate those symptoms.

### 3.3. Normal Sensory Stimulation and the Maintenance of Brain Health

If individuals with ASD need enhanced sensory stimulation to experience typical neurobehavioral responses, is it the case that neurotypical individuals also need a high level of sensory stimulation to sustain normal brain function? In fact, the loss of sensory input is associated with a decline in higher-order functioning, including both facilitating a cognitive decline in older adults and increasing the risk of intellectual disability in children [87, 88]. For example, visual impairment and hearing impairment are associated with cognitive dysfunction in humans [89–92]. Similarly, mastication problems are also associated with cognitive loss [93, 94].

Longitudinal studies of older adults without initial cognitive impairment found that the failure to identify odors predicted the onset of mild cognitive impairment within five years [95–98]. In another study of nondemented older adults, poor odor identification, along with aging and having the ApoE-4 allele, predicted an increased cognitive decline over five years that could not be predicted by performance on a vocabulary test [99]. Even within a three-year period, poor olfactory discrimination predicted a significant cognitive decline [100]. Similarly, self-reports of poor olfactory function predicted the onset of dementia over a ten-year period [101]. Among those individuals who already had mild cognitive impairment, poor olfactory abilities predicted the onset of dementia [102, 103].

Anosmic individuals experience a loss of gray matter in their medial prefrontal cortex, the subcallosal gyrus, the nucleus accumbens, the dorsolateral prefrontal cortex, cerebellum, occipital gyrus, piriform cortex, anterior insular cortex, orbital frontal cortex, supramarginal gyrus, precuneus, hippocampus, and parahippocampal region, brain areas that include those involved in cognitive function [104]. The longer the olfactory loss, the more severe the loss of gray matter in these areas. Peng et al. [105] similarly showed extensive loss of both gray and white matter in the brains of anosmics, a neural loss which was exacerbated with increased duration of the sensory loss. When hyposmic individuals who have impaired olfactory function were examined, their gray matter was diminished in the insular cortex, anterior cingulate cortex, orbitofrontal cortex, cerebellum, fusiform gyrus, precuneus, middle temporal gyrus, and piriform cortex. In addition, their white matter was diminished underneath the insular cortex, in the cerebellum, and in the middle frontal gyrus [106]. Even distorted olfactory experience in the case of paranosmia is associated with a diminishment of gray matter in a variety of brain areas [107].

While sensory loss and cognitive decline may be independent of each other, there is at least some reason to believe that their relationship can be causal: that sensory loss can speed cognitive decline. For example, diminished olfactory and auditory abilities accurately predict subsequent cognitive decline in prospective studies [100, 108]. In addition, the use of hearing aids or the provision of cochlear implants can induce cognitive gains in people with hearing loss [109, 110]. Prevention of vision loss in a mouse model of glaucoma prevented its cognitive decline [111] and active mastication improved cognitive function in humans following its diminishment with the inability to chew normally [112].

### 3.4. Animal Models of Autism Respond to Environmental Enrichment

Animal models of syndromic forms of autism have shown that enriched environments can ameliorate the autism-like symptoms that are seen under low levels of sensory stimulation. An enriched environment for experimental animals allows for increased social interactions in a large cage, along with the opportunity to engage with a variety of inanimate objects and to have the ability to exercise [113]. There is a mouse model of autism that mimics Rett syndrome, with the same gene deletion as humans who have the syndrome. When these mice are housed in an enriched sensorimotor environment, their autism-like symptoms, motor coordination, memory, and anxiety improve [114–116]. Moreover, an enriched environment normalizes the excitatory and inhibitory synaptic densities in their cerebellum and cortex [116], restores cortical long-term potentiation, increases cortical brain-derived neurotrophic factor, and improves the expression of synaptic markers [114, 116].

Fragile X syndrome results from a mutation of the FMR1 gene and often produces children with symptoms that are characteristic of autism. Sensorimotor enrichment similarly rescues FMR1 knockout mice from cognitive deficiencies, reduces anxiety, and increases their exploratory behavior [117].

Most humans with Potocki-Lupski syndrome are diagnosed with autism [118, 119] and the mouse model of this syndrome also has autism-like symptoms, including abnormal ultrasonic vocalizations, perseverative/stereotypic behaviors, anxiety, deficits in learning and memory, and motor deficits when they are housed in a low-stimulation environment [120]. Mice with this genetic anomaly living in an enriched sensorimotor environment have improved motor skills, improved learning and memory, reduced aggressive behavior, and reduced anxiety, although it did not improve their social abnormalities or their abnormal vocalizations [120]. As a whole, these data point to the conclusion that these genetic anomalies are only capable of producing their autism-like syndrome under limited environmental stimulation.

There are three other animal models of autism that also respond well to environmental enrichment. BTBR mice have been differentially bred to express what appear to be core symptoms of autism. Specifically, they have impaired social interactions, deficits in communication, poor social transmission of food preferences, and repetitive behaviors [121–123]. This model of autism also responds well to environmental enrichment, normalizing their repetitive grooming behaviors and their repetitive exploration of objects, as well as their cognitive ability [124, 125]. When BTBR mice were given social enrichment by housing them with a very social mouse strain, the BTBR mice showed improved sociability, but it did not normalize their repetitive behaviors [126].

Deer mice who are kept isolated in a small cage engage in repetitive, stereotyped behavior that resembles ASD behavioral patterns. Such behavior is normalized in an enriched environment [127].

Fetal exposure to valproic acid increases the expression of ASD symptoms in humans, and it has similar effects in rats [128]. Animals exposed to valproic acid in fetal life have a suppressed pain response, increased anxiety, hypersensitivity to sensory stimuli, repetitive and stereotyped behaviors, decreased exploration, and limited social interactions [129]. In addition, these rats have lower acoustic prepulse inhibition, which is involved in adaptation to sensory input [129]. Again, sensory enrichment in such rats ameliorated their autism-like symptoms, including decreased repetitive activity and anxiety, while increasing normal exploratory activity and social behaviors [129].

In addition to ASD, environmental enrichment is effective in ameliorating the symptoms of a large number of neurological disorders [130–134] and it seems quite possible that other neurological disorders can be treated with this approach. Indeed, we have initiated an effort to determine the efficacy of environmental enrichment for the treatment of other developmental neurobehavioral disorders.

### 3.5. Sensory Abnormalities in ASD

How might increased sensorimotor experiences ameliorate the symptoms of autism? Up to 95% of children with autism have sensory processing abnormalities that include increased sensory seeking behavior, avoidance or diminished responses to some sensory stimuli, and enhanced perceptual abilities [135–142]. Indeed, abnormal sensory reactivity is included in the current DSM-5 diagnostic criteria for ASD [143].

Some of the sensory abnormalities that have been described in ASD occur early in neural sensory processing and therefore raise the possibility that the core symptoms of ASD may be responses to abnormal sensory input [144–146]. For example, the strength of perceptual binding of audiovisual speech observed in individuals with ASD is strongly related to their low-level multisensory temporal processing abilities, suggesting that sensory problems may underlie core elements of their disorder [147, 148]. Alternatively, the anxiety evoked by abnormal sensory responses may be ameliorated by engaging in repetitive behaviors and/or rituals [149]. Indeed, anxiety in preschoolers with ASD increases the probability of their engaging in rituals [150]. Differences in temperament, personality, language, and social development of children with ASD also appear to be related to their sensory problems [151, 152]. Environmental enrichment decreases abnormal sensory responses [18] and this ability may underlie part of its effectiveness in reducing other symptoms of autism.

### 3.6. Study Limitations

While these data suggest the feasibility of a real-world online treatment for ASD using environmental enrichment, there are several limitations of this study. At the same time, it is important to point out that these data are quite consistent with the outcomes of the two randomized clinical trials that evaluated environmental enrichment for the treatment of ASD [17, 18].

Because parents self-selected the number of worksheets that they received, there are limits regarding the interpretation of these findings, as there may have been other variables associated with that behavior that may have actually caused the lower level of improvement in the children of those parents who appeared to be unengaged with the therapy.

Another factor is that most parents were paying for this therapy and financial considerations may well have been a factor in determining the length of time that parents were willing to participate in the program, or it may have affected their evaluation of the outcomes for their child. However, there was no difference in outcomes reported by parents who were paying and parents who were scholarship recipients and were not paying for the treatment. It also should be noted that an even lower-cost alternative payment plan has recently been instituted for this online therapy and this change has reduced patient dropout from the program. In addition, while finances may have been a variable in determining the length of treatment, the fact that some children experienced a rapid, large improvement in their symptoms raises the possibility that their parents may have stopped treatment because their child had made good progress on the therapy, rather than stopping due to financial reasons or dissatisfaction with the therapy.

An additional limitation of this study is that there were no professional diagnoses for the subjects. The subjects in this study had a variety of reported diagnoses or no reported diagnosis. However, diagnostic categorization of psychiatric disorders does not correlate well with the biological bases of the disorders [153], and the National Institute for Mental Health has concluded that it makes more sense to evaluate psychiatric issues based on individual symptoms, as we have done, rather than relying on diagnostic categories to describe subjects in clinical trials [153].

Parents were also the only source of information regarding the outcomes for their children. However, most assessments of treatments for ASD rely on parental feedback for the determination of symptom improvement, including the Strengths and Difficulties Questionnaire, the Vineland Adaptive Behavior Scale, the Childhood Autism Rating Scale, the Aberrant Behavior Checklist, the Short Sensory Profile, the Modified Checklist of Autism in Toddlers, the Autism Diagnostic Interview, the Social Communication Questionnaire, the Autism Behavior Checklist, the Gilliam Autism Rating Scale, the Parent Interview for Autism, the Asperger Syndrome Diagnostic Scale, the Autism Spectrum Screening Questionnaire, the PDD Behavior Inventory, the Children's Communication Checklist, and the Childhood Autism Spectrum Test. Direct observation, using, for example, both the Autism Diagnostic Observation Schedule and the Global Clinical Impression Scale, relies on limited time spent with the child under atypical conditions. These assessment tools are often inadequate, on their own, to reveal reliable changes in outcomes over time. The former test requires an evaluator to determine whether or not the subject's highly variable behavior is typical or atypical in sessions six months apart and the latter asks the assessor to compare the behavior of the child at the initiation of the therapy to the behavior shown after 6 months of therapy. The reality is that it is difficult to obtain critical information about the progress of ASD children without being able to observe their behavior on a daily basis. While the parents were the only source of assessment in this study, their conclusions were consistent with both the objective and subjective measures used in our previous two randomized clinical trials that showed improvements for children with ASD after Sensory Enrichment Therapy.

This study also did not have a control group to compare to those given Sensory Enrichment Therapy, and it is therefore possible that the benefits of this therapy may have been seen simply with the passage of time. On the other hand, the noncompliant parents who continued their assessments for at least 6 months had a much smaller improvement in their child's symptoms than compliant parents. These data suggest that there was a critical difference in the outcomes that depended on the intensity of the treatment. It is also the case that the outcomes of children treated with Sensory Enrichment Therapy appear to be much better than the developmental trajectories of 6,975 children with autism, aged 2–14, who were assessed repeatedly over a long period of time [154]. They found that children with ASD who did not have access to this therapy had heterogeneous developmental pathways. Unlike our treated children, the children that they followed with a low initial ASD severity score tended to have the greatest improvements over time, and few of the children that they followed experienced a major improvement in their symptoms, particularly over the initial 7 months.

There was also limited demographic information of subjects and their parents in our study, aside from age, gender, and geographic origin. Neither was there information collected regarding their concurrent use of pharmaceuticals, concurrent behavioral/medical treatments, or the training level of concurrent treatment providers. The diagnoses and patient age were heterogeneous, as one would expect in the real world, but that enhances the generalizability of our conclusions. In addition, while there was objective evidence of whether the parents downloaded worksheets, there was no objective assessment of the fidelity with which they administered the treatment to their children. We also do not know whether the same parent completed all of the assessments.

There is always a trade-off between the internal validity provided by well-run randomized clinical trials in evaluating the efficacy of a treatment and the external validity evaluating the effectiveness of the treatment when it is given to a broad variety of individuals under real-world circumstances. In this study, we have evaluated the effectiveness of Sensory Enrichment Therapy with a large number of diverse individuals and have shown that the efficacy previously demonstrated for this therapy in clinical trials can also be seen to be effective in the real world.

#### 3.6.1. Gene-Environment Interactions in Autism

The genetic underpinnings of autism spectrum disorder have been established with studies of twins, families, and populations [3, 155–158], but these data also make it clear that there is also a significant environmental risk for the expression of autism symptoms. The heterogeneity of both the symptoms and the genetics are high, but the phenotypic heterogeneity does not correlate well with the genetic heterogeneity [159]. There is extraordinary complexity in the underlying genetics, with hundreds of common and rare genetic variants increasing the risk for ASD, with the preponderance of risk due to common variations [158]. In addition, the total burden of these genetic variants is correlated with the expression of ASD symptoms [158, 160]. Moreover, the same single-nucleotide polymorphisms can be shared with ASD, attention-deficit hyperactivity disorder, bipolar disorder, major depressive disorder, or schizophrenia [161].

Although the early estimates from twin studies of the relative contribution of genes and environment greatly favored the role of genes in elevating ASD risk [155–157], more recent studies using genome-wide estimates have about equal risk assigned to genes and environment [3, 158]. This change in the relative importance of genes and environment may be due to a number of variables, including changes in the ways by which ASD is diagnosed, with the diagnostic category expanding to include Asperger's syndrome. Another possibility is that those individuals with syndromic ASD are less likely to be included in recent studies, as differential diagnoses of these disorders has improved. Differences in statistical modeling of the data may also have contributed to this shift.

There are a number of risk factors that suggest an interaction between genes and environment in ASD. For example, there is a strong relationship between paternal age and ASD risk [162], perhaps due to an increase in genetic anomalies with age [163]. Importantly, Hultman et al. [162] showed that it was not due to having a father who had ASD-like symptoms and was unable to find a mate earlier in life. To the contrary, they showed that, in families with one child diagnosed with ASD, that child was likely to be born to the father when he was older than when the children without autism were born. In addition, the time since the birth of one child predicted the occurrence of ASD in the other child. Advanced paternal age also predicted a higher concordance rate for ASD between both monozygotic and dizygotic twins, suggesting that the additional genetic anomalies that come with advancing paternal age may add to other genetic anomalies to result in an ASD diagnosis [164]. Finally, Frans et al. [165] showed that increasing age of the grandfather also predicted increased risk for ASD in the grandchildren, suggesting that environmental experiences well in advance of the child's conception appear to increase the risk of ASD.

Older mothers also have children with an increased ASD risk [166], even where various other factors involved with pregnancy and birth are considered. While younger mothers have eggs that respond to DNA damage by arresting at metaphase of the first meiosis, thereby preventing abnormal embryos, older mothers have a reduced ability to engage this developmental control point and therefore are more likely to have increased chromosomal anomalies in embryos [167]. Such anomalies may result in an increased risk of ASD.

In another example of gene x environment interaction, valproic acid has been given to pregnant women for the treatment of epilepsy, migraine, or bipolar disorder. This drug inhibits histone deacetylase, which impacts gene transcription [168], and it induces DNA demethylation [169], which dysregulates the Wnt/b-catenin signaling pathway that is involved in brain development [170]. There is also an increased risk of ASD in their children [170]. Recall that when fetal rats are exposed to valproic acid, they develop autism-like symptoms that are greatly ameliorated by living in an enriched sensorimotor environment [114–116]. These data show that the probability of expressing autism symptoms can be increased or decreased, depending on environmental experiences. Decreased sensory stimulation, along with valproic acid exposure, increases the expression of ASD symptoms and increased sensory stimulation decreases the expression of those symptoms in the animal model.

There are other environmental factors during fetal life that can increase the risk of ASD. A clear example of a gene x environment interaction can be found in the study of the risk of ASD with exposure to air pollution. Specifically, children who were exposed to high levels of air pollution either during pregnancy or as infants are at increased risk for ASD [171–174]. Air pollution appears to interact with the MET receptor tyrosine kinase gene, which is involved in mediating brain development. A variant of this gene that disrupts MET transcription is associated with an increased risk of ASD [175] and children exposed to high levels of air pollution only had an elevated risk for ASD if they also had this genetic variant [176].

## 4. Conclusions

Environmental enrichment in the form of Sensory Enrichment Therapy provided online shows promise as an effective approach for treatment of a wide range of symptoms in individuals with autism. This therapy appears to be an effective, low-cost means of treating ASD symptoms and associated symptoms across different ages, geographic location, gender, and symptom severity under real-world conditions.

## Figures and Tables

**Table 1:** Specific questions for parental assessment.

Questions regarding symptom severity	% affected	Initial score	Change
*Social skills*			
*Basic skill*			
Sitting still and waiting	89%	2.02	0.49
Eye contact	84%	2.46	0.59
Waiting for a turn	82%	2.45	0.50
Interrupting a lot	80%	2.03	0.35
Acknowledging people around him/her	75%	2.50	0.62
Ability to entertain himself/herself	51%	2.45	0.51
*Complex skill*			
Making friends	93%	1.38	0.36
Seems unable to pick up common social cues	93%	1.55	0.30
Playing with other persons of the same age	92%	1.45	0.43
Awkward in social situations	88%	1.88	0.30
Can remember people's names	61%	2.37	0.47
Inappropriate signs of affection to loved ones	51%	2.55	0.41
Showing inappropriate signs of affection to strangers	49%	2.54	0.34
Can remember people's faces	45%	3.14	0.43
Lying or stealing	25%	3.03	0.49
Making threats	23%	2.57	0.43
*Personality trait*			
Stubborn, cannot let it go	74%	2.35	0.33
Inflexible opinions	73%	2.16	0.39
Seeking attention	72%	2.35	0.33
Sharing toys	71%	2.50	0.38
Seems to not think before speaking	70%	2.43	0.31
Obsessed with being in control	62%	2.38	0.27
Shy	59%	2.67	0.41
Being a sore loser	54%	2.40	0.45
Suspicious or mistrusting of others	36%	2.88	0.30

*Attention span*			
*Basic skill*			
Completes instructions	84%	2.13	0.51
Attention span	84%	2.02	0.48
Can keep focus	83%	1.88	0.44
Able to concentrate	83%	2.09	0.51
Squirms or fidgets	82%	2.00	0.41
Pacing	61%	2.19	0.40
*Complex skill*			
Needs reminders	85%	1.81	0.33
Cannot sit through something boring	83%	1.82	0.36
Gets bored easily	83%	2.27	0.40
Planning ahead	83%	1.46	0.40
Finishes what they started	82%	1.77	0.40
Can finish lengthy projects	82%	1.29	0.32
Organizing self for an activity	82%	1.51	0.45
Independently prepares for things	81%	1.45	0.44
Cannot sit still	81%	2.09	0.41
Accomplishes complicated tasks	80%	1.63	0.36
Absent-minded	79%	1.83	0.43
Constantly on the move	78%	2.00	0.38
Loses or misplaces things	73%	2.09	0.37
Has trouble deciding things	71%	2.18	0.48

*Communication*			
*Basic skill*			
Sharing thoughts with words	83%	1.44	0.56
Speaking in sentences	79%	1.34	0.48
Pronunciation	75%	1.82	0.49
Vocabulary	75%	1.53	0.51
Repeating things over and over	70%	1.93	0.35
Communicating needs with or without words	66%	2.56	0.63
Uses the wrong words for things	57%	2.54	0.35
Responding to his/her name	46%	2.96	0.55
Stuttering	18%	2.41	0.48
*Complex skill*			
Understands what others are saying	74%	2.64	0.59
Seems to just repeat what he/she heard	69%	2.08	0.43
Uncontrolled swearing	9%	2.26	0.33

*Learning*			
*Basic skill*			
Dressing self	66%	2.47	0.53
Learning new concepts	62%	2.40	0.62
Relating things together	61%	2.38	0.60
Identifying patterns	53%	2.33	0.47
Knows numbers	31%	2.22	0.49
Knows the alphabet letters	27%	1.96	0.51
Knows colors	24%	2.34	0.50
*Complex skill*			
Retells stories	78%	1.39	0.33
Understands what is going on in a story	77%	2.01	0.42
Follows a plot	73%	1.71	0.29
Reads with expression	72%	1.55	0.28
Tying shoelaces	71%	1.27	0.23
Guesses words instead of sounding them out	65%	1.76	0.30
Can learn abstract concepts	64%	1.61	0.41
Understands concepts of time	60%	1.67	0.43
Enjoys being read to	60%	2.14	0.40
Understands denominations of money have different value	60%	1.49	0.36
Basic math skills	55%	1.85	0.42
Spelling	54%	1.84	0.41
Understands what is not seen still exists	45%	2.19	0.47

*Mood & behavior*			
*Basic skill*			
Giving in to cravings	74%	2.32	0.30
Cannot interrupt favorite activities	71%	2.68	0.45
Frequency of tantrums	70%	2.87	0.44
Expressing emotion	66%	2.55	0.49
Duration of tantrums	59%	2.94	0.50
Unexplained bursts of laughter	59%	2.59	0.32
Thrashing	43%	2.67	0.52
Low energy levels	38%	2.82	0.50
Hates spills on their clothes	37%	2.81	0.47
Aggressive toward self	37%	2.85	0.43
Cannot be away from primary caregiver	32%	2.93	0.53
*Complex skill*			
Severity of tantrums	66%	2.78	0.46
Feels like mind is in a fog	62%	2.47	0.44
Seems obsessed with one topic	58%	2.43	0.36
Unreasonable fears	42%	2.96	0.39
Unable to discard broken or worthless things	36%	2.70	0.50
Cannot part with favorite blanket or object	27%	2.88	0.49
Compulsive spending	25%	2.49	0.49
Preoccupied with germs	9%	2.91	0.58
Suicidal thoughts/side effects	5%	3.41	0.75
*Personality trait*			
Impulsive	79%	2.05	0.29
Shouting instead of verbalizing	66%	2.35	0.34
Feeling appropriate emotions	66%	2.77	0.38
Getting overexcited easily	65%	2.57	0.30
Screaming and screeching	62%	2.35	0.38
Becoming discouraged easily	62%	2.57	0.35
Ability to relax	62%	2.62	0.42
Whining and complaining	59%	2.67	0.30
Feeling serene	59%	2.72	0.38
Crying	57%	2.96	0.35
Aggressive toward others	55%	2.88	0.38
Gets angry quickly and a lot	53%	2.79	0.38
Cannot snap out of a bad mood	43%	3.01	0.43
Regularly changes between overenthusiastic and miserable	42%	2.86	0.37
Tendency to feel depressed	33%	3.08	0.37
Obsessed with perfection	29%	2.98	0.41
Panic attacks	25%	3.03	0.36
Feeling guilty for no real reason	22%	3.16	0.33
Preoccupied with tidiness	20%	3.04	0.37
Obsession with death	10%	3.26	0.29

*Anxiety*			
*Basic skill*			
Repetitive mannerisms	73%	2.20	0.32
Watching the same show over and over	70%	2.01	0.42
Repetitive motion all the time	61%	2.33	0.36
Flaps hands when excited	59%	2.20	0.47
Repetitive motion can be interrupted	51%	2.96	0.45
Taps, clicks, pops, sniffs, or other tics	43%	2.37	0.43
Grinding teeth	42%	2.58	0.67
Twitches or other motor tics	35%	2.65	0.36
Rocking back and forth	28%	2.68	0.55

*Sensory processing*			
*Basic skill*			
Sensitive to loudness	78%	2.35	0.47
Sensitive to busy loud crowds	76%	2.25	0.50
Cannot handle transitions	75%	2.58	0.50
Does not prepare for cold or warm	68%	2.38	0.44
Sensitive to certain tastes	63%	2.64	0.38
Accepts to wear a blindfold	60%	2.18	0.48
Sensitive to electric motor sound	59%	2.41	0.51
Sensitive to certain voices	57%	2.47	0.42
Sensitive to certain textures	57%	2.81	0.57
Hates brushing his/her teeth	53%	2.57	0.60
Seems insensitive to cold	48%	2.77	0.51
Bothered by water in his/her ears	48%	2.68	0.53
Sensitive to clothing	46%	2.86	0.52
Sensitive to light	45%	2.98	0.44
Does not seem to feel pain	44%	2.89	0.56
Sensitive to certain smells	43%	2.98	0.46
Sensitive when touched	42%	3.01	0.50
Quality of the sense of smell	40%	3.22	0.63
Sensitive to feeling motion	37%	2.98	0.48
Can discern different flavors	36%	3.13	0.53
Can breathe in deeply	33%	2.97	0.54
Hates wearing shoes	32%	2.80	0.66
Sensitive to moving objects	32%	3.12	0.47
Sensitive to dark	31%	3.06	0.43
Ability to detect sounds	28%	3.16	0.52
Looks flushed and overheated	26%	3.25	0.44
Can feel and locate light touch	26%	3.23	0.54
Sensitive to silence	19%	3.17	0.44
Experiences unexplained tingling sensation	16%	3.50	0.06
Can see well (with vision aids if needed)	14%	3.41	0.34
Sweating suddenly for no reason	13%	3.08	0.54
*Complex skill*			
Seems unaware of threatening situations	75%	2.21	0.45
Sensitive to certain pitches	72%	2.31	0.38
Hates having haircuts	64%	2.13	0.56
Scared of heights	42%	3.01	0.43
Hears things that are not there	19%	3.08	0.48
Sees things that are not there	19%	3.10	0.51

*Eating*			
*Basic skill*			
Tries new foods	62%	1.92	0.57
Tolerates different food textures	62%	2.15	0.43
Leaves dinner table	60%	2.17	0.34
Use of utensils	59%	2.56	0.42
Eats a variety of foods	58%	2.05	0.46
Accepts food	45%	2.47	0.58
Consistency of BM	40%	2.70	0.46
Regular bowel movements	36%	2.72	0.52
Gagging	34%	2.66	0.59
Flatulence	33%	2.98	0.33
Eats too little	30%	2.55	0.44
Can feed himself/herself	28%	2.93	0.51
Eats too much	22%	2.93	0.34
Ability to chew	20%	2.88	0.56
Ability to swallow	13%	3.15	0.58

*Self-awareness*			
*Basic skill*			
Imaginary play	61%	1.96	0.53
Unaware of surroundings	57%	2.76	0.55
Unaware of self	53%	2.82	0.50
Talking to himself/herself	52%	2.32	0.36
Bladder control at night	44%	1.94	0.51
Daytime bladder control	34%	2.02	0.64
Daytime bowel movement control	34%	1.76	0.63
Does not want to shower or bathe	31%	2.83	0.55
Bowel movement control at night	23%	1.71	0.58
*Complex skill*			
Grooming and caring for appearing neat	68%	2.32	0.26
Things do not seem real to him/her	35%	3.07	0.30
Experiences “déja-vu”	26%	2.76	0.41
Real life seems like it is a dream	26%	3.05	0.30
Talking to people who are not there	19%	2.68	0.45
*Personality trait*			
Daydreaming	53%	2.52	0.28

*Memory*			
*Basic skill*			
Can remember instructions	54%	2.22	0.45
Can remember what happened yesterday	42%	2.25	0.47
Visual memory	32%	2.99	0.37
*Complex skill*			
Can give directions to where they put something	50%	1.42	0.33
Can remember directions to go find something	46%	2.17	0.43
Can remember dates	44%	1.43	0.34
Can remember facts	43%	1.91	0.40
Can remember events	40%	2.15	0.41
Can remember important events years ago	38%	1.86	0.29
Can remember sequence of numbers	36%	2.29	0.38
Can remember sequence of letters	34%	2.17	0.37
Can remember songs	28%	2.83	0.53

*Motor skills*			
*Basic skill*			
Balance on the left leg	53%	2.57	0.45
Balance on the right leg	52%	2.58	0.43
Balance in general	44%	2.95	0.54
Tongue control	44%	2.69	0.45
Accident prone	43%	2.86	0.43
Walks into things	43%	2.87	0.47
Tripping	41%	3.01	0.48
Falling	36%	3.05	0.50
Can keep his/her eyes on a moving target	35%	3.16	0.41
Muscles are limp	31%	2.76	0.53
Can scan with his/her eyes, left to right, top to bottom	31%	3.10	0.46
Jumping	30%	2.69	0.48
Strength of right arm	28%	3.01	0.43
Strength of left arm	28%	3.03	0.43
Walks on tippy toes	27%	2.45	0.51
Control of fingers of left hand	26%	2.89	0.49
Control of fingers of right hand	26%	2.89	0.51
Strength of right leg	26%	3.08	0.42
Strength of left leg	26%	3.10	0.44
Running	22%	3.01	0.38
Strength of neck	21%	3.21	0.43
Involuntary movement occurs in the body in general	20%	2.93	0.58
Right leg muscles are always tight	20%	2.92	0.43
Left leg muscles are always tight	19%	2.94	0.44
Control of right hand	19%	3.11	0.46
Control of left hand	19%	3.08	0.47
Drooling	17%	2.92	0.62
Control of right leg	16%	3.10	0.51
Control of left leg	16%	3.10	0.51
Can go downstairs	16%	2.91	0.59
Control of right arm	15%	3.09	0.49
Control of left arm	15%	3.15	0.45
Right arm muscles are always tight	14%	3.24	0.41
Left arm muscles are always tight	14%	3.28	0.41
Involuntary movements occur in fingers of right hand	14%	2.93	0.50
Involuntary movements occur in fingers of left hand	13%	2.95	0.49
Involuntary movements occur in right hand	13%	2.95	0.52
Involuntary movements occur in left hand	13%	3.03	0.57
Can go upstairs	12%	2.95	0.70
Control of neck	12%	3.12	0.47
Involuntary movements occur in right arm	12%	3.03	0.55
Involuntary movements occur in left arm	12%	3.10	0.53
Involuntary movements occur in right leg	10%	3.22	0.55
Involuntary movements occur in left leg	10%	3.22	0.54
Sitting up	10%	3.03	0.56
Shakes all the time	9%	3.14	0.58
Crawling	8%	2.96	0.49
Walking	6%	2.89	0.63
Standing on own	4%	2.36	0.61
Standing being supported	3%	2.97	0.79
*Complex skill*			
Writing penmanship	77%	1.73	0.44
Stays in the lines when coloring	74%	1.74	0.41
Drawing ability	73%	1.85	0.46
Scissor control	71%	2.23	0.43
Catching with one hand	66%	1.89	0.27
Catching with two hands	58%	2.47	0.45
Ability to do push-ups	57%	2.13	0.25
Clumsiness	56%	2.74	0.45
Throwing skill	55%	2.70	0.44
Ability to do sit-ups	51%	2.26	0.24
Riding a bicycle	49%	1.69	0.36
Control of facial expression	48%	2.78	0.39
Kicking a ball	48%	2.78	0.40
Posture	45%	2.91	0.37
Ability to do squats	44%	2.51	0.30
Strength of left hand	37%	2.89	0.40
Strength of right hand	36%	2.86	0.43
Riding a tricycle	36%	2.12	0.44
Climbing skills	32%	2.97	0.47
Control of right ankle	17%	3.03	0.41
Control of left ankle	17%	3.02	0.42

*Sleep*			
*Basic skill*			
Falls asleep right away	53%	2.55	0.41
Has difficulty going back to sleep	48%	2.66	0.48
Will not stay asleep	43%	2.75	0.46
Wakes up grumpy	34%	3.04	0.55
Sleeps in own bed	30%	2.22	0.46
Sleeps in	28%	2.85	0.43
Wakes up screaming at night	18%	3.21	0.61
Falls asleep unexpectedly	7%	3.20	0.57
*Complex skill*			
Has bad dreams	29%	3.41	0.44

*Other*			
Is in physical pain	17%	3.27	0.19
Frequency of absence episodes	5%	3.22	0.79
Duration of absence episode	5%	3.57	0.47
Difficulty to interrupt an absence episode	4%	3.03	0.77
Frequency of convulsions	3%	2.93	0.77
Intensity of convulsions	3%	3.24	0.80
Unexplained body stiffening episodes	3%	3.24	0.22
Duration of convulsions	2%	3.57	0.62
Recovery time after an absence episode	2%	3.32	0.74
Recovery time after a convulsion	2%	3.53	0.57
Unexplained eye-rolling episodes	2%	3.18	0.68
Unexpected loss of muscle tone	1%	2.86	0.76
Feeling outside of body	1%	3.82	−0.30
Unexplained buzzing feeling	1%	3.71	−0.43
Unexpected loss of consciousness	0%	3.25	0.47
Unexpected blackouts	0%	3.50	1.00

## Appendix

See Table 1.

## References

[1] Zablotsky B., Black L. I., Maenner M. J., Schieve L. A., Blumberg S. J. (2015). Estimated prevalence of autism and other developmental disabilities following questionnaire; Changes in the 2014 National Health Interview Survey. *National Health Statistics Report*.

[2] Colvert E., Tick B., McEwen F. (2015). Heritability of autism spectrum disorder in a UK population-based twin sample. *JAMA Psychiatry*.

[3] Sandin S., Lichtenstein P., Kuja-Halkola R., Larsson H., Hultman C. M., Reichenberg A. (2014). The familial risk of autism. *The Journal of the American Medical Association*.

[4] Tick B., Bolton P., Happé F., Rutter M., Rijsdijk F. (2015). Heritability of autism spectrum disorders: a meta-analysis of twin studies. *Journal of Child Psychology and Psychiatry*.

[5] Lavelle T. A., Weinstein M. C., Newhouse J. P., Munir K., Kuhlthau K. A., Prosser L. A. (2014). Economic burden of childhood autism spectrum disorders. *Pediatrics*.

[6] Klein N., Kemper K. J. (2016). Integrative approaches to caring for children with autism. *Current Problems in Pediatric and Adolescent Health Care*.

[7] Benevides T. W., Carretta H. J., Lane S. J. (2016). Unmet need for therapy among children with autism spectrum disorder: results from the 2005-2006 and 2009-2010 national survey of children with special health care needs. *Maternal and Child Health Journal*.

[8] Pennington R., Horn C., Berrong A. (2009). An evaluation of the differences between big city and small town special education services for students with low incidence disabilities in Kentucky. *Rural Special Education Quarterly*.

[9] Ganz M. L. (2007). The lifetime distribution of the incremental societal costs of autism. *Archives of Pediatrics and Adolescent Medicine*.

[10] MacDonald R., Parry-Cruwys D., Dupere S., Ahearn W. (2014). Assessing progress and outcome of early intensive behavioral intervention for toddlers with autism. *Research in Developmental Disabilities*.

[11] Steinhausen H.-C., Mohr Jensen C., Lauritsen M. B. (2016). A systematic review and meta-analysis of the long-term overall outcome of autism spectrum disorders in adolescence and adulthood. *Acta Psychiatrica Scandinavica*.

[12] Eikeseth S., Smith T., Jahr E., Eldevik S. (2002). Intensive behavioral treatment at school for 4- to 7-year-old children with autism. A 1-year comparison controlled study. *Behavior Modification*.

[13] Goldstein H. (2002). Communication intervention for children with autism: a review of treatment efficacy. *Journal of Autism and Developmental Disorders*.

[14] Case-Smith J., Arbesman M. (2008). Evidence-based review of interventions for autism used in or of relevance to occupational therapy. *American Journal of Occupational Therapy*.

[15] Bitterman A., Daley T. C., Misra S., Carlson E., Markowitz J. (2008). A national sample of preschoolers with autism spectrum disorders: special education services and parent satisfaction. *Journal of Autism and Developmental Disorders*.

[16] Reynolds S., Lane S. J., Richards L. (2010). Using animal models of enriched environments to inform research on sensory integration intervention for the rehabilitation of neurodevelopmental disorders. *Journal of Neurodevelopmental Disorders*.

[17] Woo C. C., Leon M. (2013). Environmental enrichment as an effective treatment for autism: a randomized controlled trial. *Behavioral Neuroscience*.

[18] Woo C. C., Donnelly J. H., Steinberg-Epstein R., Leon M. (2015). Environmental enrichment as a therapy for autism: a clinical trial replication and extension. *Behavioral Neuroscience*.

[19] Dunn W. (1999). *The Sensory Profile Manual*.

[20] Hamad C. D., Serna R. W., Morrison L., Fleming R. (2010). Extending the reach of early intervention training for practitioners: a preliminary investigation of an online curriculum for teaching behavioral intervention knowledge in autism to families and service providers. *Infants & Young Children*.

[21] Hepburn S. L., Blakeley-Smith A., Wolff B., Reaven J. A. (2016). Telehealth delivery of cognitive-behavioral intervention to youth with autism spectrum disorder and anxiety: a pilot study. *Autism*.

[22] Ingersoll B., Berger N. I. (2015). Parent engagement with a telehealth-based parent-mediated intervention program for children with autism spectrum disorders: predictors of program use and parent outcomes. *Journal of Medical Internet Research*.

[23] Jang J., Dixon D. R., Tarbox J., Granpeesheh D., Kornack J., de Nocker Y. (2012). Randomized trial of an eLearning program for training family members of children with autism in the principles and procedures of applied behavior analysis. *Research in Autism Spectrum Disorders*.

[24] Lindgren S., Wacker D., Suess A. (2016). Telehealth and autism: treating challenging behavior at lower cost. *Pediatrics*.

[25] Nefdt N., Koegel R., Singer G., Gerber M. (2010). The use of a self-directed learning program to provide introductory training in pivotal response treatment to parents of children with autism. *Journal of Positive Behavior Interventions*.

[26] Vismara L. A., Young G. S., Rogers S. J. (2012). Telehealth for expanding the reach of early autism training to parents. *Autism Research and Treatment*.

[27] Vismara L. A., McCormick C., Young G. S., Nadhan A., Monlux K. (2013). Preliminary findings of a telehealth approach to parent training in autism. *Journal of Autism and Developmental Disorders*.

[28] Wainer A. L., Ingersoll B. R. (2015). Increasing access to an ASD imitation intervention via a telehealth parent training program. *Journal of Autism and Developmental Disorders*.

[29] Carter A. S., Messinger D. S., Stone W. L., Celimli S., Nahmias A. S., Yoder P. (2011). A randomized controlled trial of Hanen's ‘More Than Words’ in toddlers with early autism symptoms. *Journal of Child Psychology and Psychiatry*.

[30] Rogers S. J., Estes A., Lord C. (2012). Effects of a brief early start Denver model (ESDM)-based parent intervention on toddlers at risk for autism spectrum disorders: a randomized controlled trial. *Journal of the American Academy of Child and Adolescent Psychiatry*.

[31] Schertz H. H., Odom S. L., Baggett K. M., Sideris J. H. (2013). Effects of Joint Attention Mediated Learning for toddlers with autism spectrum disorders: an initial randomized controlled study. *Early Childhood Research Quarterly*.

[32] Siller M., Sigman M. (2002). The behaviors of parents of children with autism predict the subsequent development of their children's communication. *Journal of Autism and Developmental Disorders*.

[33] McConachie H., Diggle T. (2007). Parent implemented early intervention for young children with autism spectrum disorder: a systematic review. *Journal of Evaluation in Clinical Practice*.

[34] Oono I. P., Honey E. J., McConachie H. (2013). Parent-mediated early intervention for young children with autism spectrum disorders (ASD). *The Cochrane Database of Systematic Reviews*.

[35] Leyfer O. T., Folstein S. E., Bacalman S. (2006). Comorbid psychiatric disorders in children with autism: interview development and rates of disorders. *Journal of Autism and Developmental Disorders*.

[36] Maski K. P., Jeste S. S., Spence S. J. (2011). Common neurological co-morbidities in autism spectrum disorders. *Current Opinion in Pediatrics*.

[37] Posserud M., Hysing M., Helland W., Gillberg C., Lundervold A. J. (2016). Autism traits: the importance of ‘co-morbid’ problems for impairment and contact with services. Data from the Bergen Child Study. *Research in Developmental Disabilities*.

[38] Peters-Scheffer N., Didden R., Korzilius H., Sturmey P. A. (2011). A meta-analytic study on the effectiveness of comprehensive ABA-based early intervention programs for children with autism spectrum disorders. *Research in Autism Spectrum Disorders*.

[39] Weitlauf A. S., McPheeters M. L., Peters B. (2014). *Therapies for Children With Autism Spectrum Disorder: Behavioral Interventions Update*.

[40] Brown R., Hobson R. P., Lee A., Stevenson J. (1997). Are there ‘autistic-like’ features in congenitally blind children?. *Journal of Child Psychology and Psychiatry and Allied Disciplines*.

[41] Mukaddes N. M., Kilincaslan A., Kucukyazici G., Sevketoglu T., Tuncer S. (2007). Autism in visually impaired individuals. *Psychiatry and Clinical Neurosciences*.

[42] Steffenburg S. (1991). Neuropsychiatric assessment of children with autism: a population-based study. *Developmental Medicine and Child Neurology*.

[43] Robertson C. E., Thomas C., Kravitz D. J. (2014). Global motion perception deficits in autism are reflected as early as primary visual cortex. *Brain*.

[44] Pei F., Baldassi S., Norcia A. M. (2014). Electrophysiological measures of low-level vision reveal spatial processing deficits and hemispheric asymmetry in autism spectrum disorder. *Journal of Vision*.

[45] Donaldson A. I., Heavner K. S., Zwolan T. A. (2004). Measuring progress in children with autism spectrum disorder who have cochlear implants. *Archives of Otolaryngology—Head and Neck Surgery*.

[46] Daneshi A., Hassanzadeh S. (2007). Cochlear implantation in prelingually deaf persons with additional disability. *The Journal of Laryngology & Otology*.

[47] Rosenhall U., Nordin V., Sandström M., Ahlsén G., Gillberg C. (1999). Autism and hearing loss. *Journal of Autism and Developmental Disorders*.

[48] Johansson M., Wentz E., Fernell E., Strömland K., Miller M. T., Gillberg C. (2001). Autistic spectrum disorders in Möbius sequence: a comprehensive study of 25 individuals. *Developmental Medicine and Child Neurology*.

[49] Johansson M., Råstam M., Billstedt E. (2006). Autism spectrum disorders and underlying brain pathology in CHARGE association. *Developmental Medicine and Child Neurology*.

[50] Johansson M., Gillberg C., Råstam M. (2010). Autism spectrum conditions in individuals with Möbius sequence, CHARGE syndrome and oculo-auriculo-vertebral spectrum: diagnostic aspects. *Research in Developmental Disabilities*.

[51] Hartshorne T. S., Grialou T. L., Parker K. R. (2005). Autistic-like behavior in CHARGE syndrome. *American Journal of Medical Genetics Part A*.

[52] Johansson M., Billstedt E., Danielsson S. (2007). Autism spectrum disorder and underlying brain mechanism in the oculoauriculovertebral spectrum. *Developmental Medicine and Child Neurology*.

[53] Foss-Feig J. H., Kwakye L. D., Cascio C. J. (2010). An extended multisensory temporal binding window in autism spectrum disorders. *Experimental Brain Research*.

[54] Kwakye L. D., Foss-Feig J. H., Cascio C. J., Stone W. L., Wallace M. T. (2011). Altered auditory and multisensory temporal processing in autism spectrum disorders. *Frontiers in Integrative Neuroscience*.

[55] Brandwein A. B., Foxe J. J., Butler J. S. (2015). Neurophysiological indices of atypical auditory processing and multisensory integration are associated with symptom severity in autism. *Journal of Autism and Developmental Disorders*.

[56] Brandwein A. B., Foxe J. J., Butler J. S. (2013). The development of multisensory integration in high-functioning autism: high-density electrical mapping and psychophysical measures reveal impairments in the processing of audiovisual inputs. *Cerebral Cortex*.

[57] Chang Y.-S., Owen J. P., Desai S. S. (2014). Autism and sensory processing disorders: shared white matter disruption in sensory pathways but divergent connectivity in social-emotional pathways. *PLoS ONE*.

[58] Hoksbergen R., ter Laak J., Rijk K., van Dijkum C., Stoutjesdijk F. (2005). Post-institutional autistic syndrome in Romanian adoptees. *Journal of Autism and Developmental Disorders*.

[59] Ellis B. H., Fisher P. A., Zaharie S. (2004). Predictors of disruptive behavior, developmental delays, anxiety, and affective symptomatology among institutionally reared Romanian children. *Journal of the American Academy of Child and Adolescent Psychiatry*.

[60] Levin A. R., Zeanah C. H., Fox N. A., Nelson C. A. (2014). Motor outcomes in children exposed to early psychosocial deprivation. *The Journal of Pediatrics*.

[61] Miller L., Chan W., Comfort K., Tirella L. (2005). Health of children adopted from Guatemala: comparison of orphanage and foster care. *Pediatrics*.

[62] Wismer Fries A. B., Ziegler T. E., Kurian J. R., Jacoris S., Pollak S. D. (2005). Early experience in humans is associated with changes in neuropeptides critical for regulating social behavior. *Proceedings of the National Academy of Sciences of the United States of America*.

[63] Colvert E., Rutter M., Kreppner J. (2008). Do theory of mind and executive function deficits underlie the adverse outcomes associated with profound early deprivation?: findings from the English and Romanian adoptees study. *Journal of Abnormal Child Psychology*.

[64] Bachevalier J., Loveland K. A. (2006). The orbitofrontal-amygdala circuit and self-regulation of social-emotional behavior in autism. *Neuroscience and Biobehavioral Reviews*.

[65] Baron-Cohen S., Ring H. A., Bullmore E. T., Wheelwright S., Ashwin C., Williams S. C. R. (2000). The amygdala theory of autism. *Neuroscience and Biobehavioral Reviews*.

[66] Baron-Cohen S., Ring H. A., Wheelwright S. (1999). Social intelligence in the normal and autistic brain: an fMRI study. *European Journal of Neuroscience*.

[67] Chugani H. T., Behen M. E., Muzik O., Juhász C., Nagy F., Chugani D. C. (2001). Local brain functional activity following early deprivation: a study of postinstitutionalized Romanian orphans. *NeuroImage*.

[68] Eluvathingal T. J., Chugani H. T., Behen M. E. (2006). Abnormal brain connectivity in children after early severe socioemotional deprivation: a diffusion tensor imaging study. *Pediatrics*.

[69] Aoki Y., Abe O., Nippashi Y., Yamasue H. (2013). Comparison of white matter integrity between autism spectrum disorder subjects and typically developing individuals: a meta-analysis of diffusion tensor imaging tractography studies. *Molecular Autism*.

[70] Pardini M., Elia M., Garaci F. G. (2012). Long-term cognitive and behavioral therapies, combined with augmentative communication, are related to uncinate fasciculus integrity in autism. *Journal of Autism and Developmental Disorders*.

[71] Pardini M., Garaci F. G., Bonzano L. (2009). White matter reduced streamline coherence in young men with autism and mental retardation. *European Journal of Neurology*.

[72] Carver L. J., Dawson G. (2002). Development and neural bases of face recognition in autism. *Molecular Psychiatry*.

[73] McCleery J. P., Akshoomoff N., Dobkins K. R., Carver L. J. (2009). Atypical face versus object processing and hemispheric asymmetries in 10-month-old infants at risk for autism. *Biological Psychiatry*.

[74] McPartland J. C., Dawson G., Webb S. J., Panagiotides H., Carver L. J. (2004). Event-related brain potentials reveal anomalies in temporal processing of faces in autism spectrum disorder. *Journal of Child Psychology and Psychiatry*.

[75] Parker S. W., Nelson C. A., Zeanah C. H. (2005). An event-related potential study of the impact of institutional rearing on face recognition. *Development and Psychopathology*.

[76] Senju A., Tojo Y., Yaguchi K., Hasegawa T. (2005). Deviant gaze processing in children with autism: an ERP study. *Neuropsychologia*.

[77] Boecker R., Holz N. E., Buchmann A. F. (2014). Impact of early life adversity on reward processing in young adults: EEG-fMRI results from a prospective study over 25 years. *PLoS ONE*.

[78] Delmonte S., Balsters J. H., McGrath J. (2012). Social and monetary reward processing in autism spectrum disorders. *Molecular Autism*.

[79] Kohls G., Schulte-Rüther M., Nehrkorn B. (2013). Reward system dysfunction in autism spectrum disorders. *Social Cognitive and Affective Neuroscience*.

[80] Mehta M. A., Gore-Langton E., Golembo N., Colvert E., Williams S. C. R., Sonuga-Barke E. (2010). Hyporesponsive reward anticipation in the basal ganglia following severe institutional deprivation early in life. *Journal of Cognitive Neuroscience*.

[81] Scott-Van Zeeland A. A., Dapretto M., Ghahremani D. G., Poldrack R. A., Bookheimer S. Y. (2010). Reward processing in autism. *Autism Research*.

[82] Bakermans-Kranenburg M. J., van Ijzendoorn M. H., Juffer F. (2008). Earlier is better: a meta-analysis of 70 years of intervention improving cognitive development in institutionalized children. *Monographs of the Society for Research in Child Development*.

[83] Bos K., Zeanah C. H., Fox N. A., Drury S. S., McLaughlin K. A., Nelson C. A. (2011). Psychiatric outcomes in young children with a history of institutionalization. *Harvard Review of Psychiatry*.

[84] Nelson C. A., Zeanah C. H., Fox N. A., Marshall P. J., Smyke A. T., Guthrie D. (2007). Cognitive recovery in socially deprived young children: the Bucharest early intervention project. *Science*.

[85] Petrenko C. L. M. (2013). A review of intervention programs to prevent and treat behavioral problems in young children with developmental disabilities. *Journal of Developmental and Physical Disabilities*.

[86] Vanderwert R. E., Marshall P. J., Nelson C. A., Zeanah C. H., Fox N. A. (2010). Timing of intervention affects brain electrical activity in children exposed to severe psychosocial neglect. *PLoS ONE*.

[87] Kiani R., Miller H. (2010). Sensory impairment and intellectual disability. *Advances in Psychiatric Treatment*.

[88] Pinto J. M., Kern D. W., Wroblewski K. E., Chen R. C., Schumm L. P., McClintock M. K. (2014). Sensory function: insights from wave 2 of the national social life, health, and aging project. *Journals of Gerontology, Series B: Psychological Sciences and Social Sciences*.

[89] Gallacher J., Ilubaera V., Ben-Shlomo Y. (2012). Auditory threshold, phonologic demand, and incident dementia. *Neurology*.

[90] Lin F. R., Yaffe K., Xia J. (2013). Hearing loss and cognitive decline in older adults. *JAMA Internal Medicine*.

[91] Moore D. R., Edmondson-Jones M., Dawes P. (2014). Relation between speech-in-noise threshold, hearing loss and cognition from 40–69 years of age. *PLoS ONE*.

[92] van Splunder J., Stilma J. S., Bernsen R. M. D., Evenhuis H. M. (2006). Prevalence of visual impairment in adults with intellectual disabilities in the Netherlands: cross-sectional study. *Eye*.

[93] Weijenberg R. A. F., Lobbezoo F. (2015). Chew the pain away: oral habits to cope with pain and stress and to stimulate cognition. *BioMed Research International*.

[94] Chen H., Iinuma M., Onozuka M., Kubo K.-Y. (2015). Chewing maintains hippocampus-dependent cognitive function. *International Journal of Medical Sciences*.

[95] Graves A. B., Bowen J. D., Rajaram L. (1999). Impaired olfaction as a marker for cognitive decline: interaction with apolipoprotein E *ε*4. *Neurology*.

[96] Swan G. E., Carmelli D. (2002). Impaired olfaction predicts cognitive decline in nondemented older adults. *Neuroepidemiology*.

[97] Wilson R. S., Schneider J. A., Arnold S. E., Tang Y., Boyle P. A., Bennett D. A. (2007). Olfactory identification and incidence of mild cognitive impairment in older age. *Archives of General Psychiatry*.

[98] Schubert C. R., Carmichael L. L., Murphy C., Klein B. E. K., Klein R., Cruickshanks K. J. (2008). Olfaction and the 5-year incidence of cognitive impairment in an epidemiological study of older adults. *Journal of the American Geriatrics Society*.

[99] Olofsson J. K., Rönnlund M., Nordin S., Nyberg L., Nilsson L.-G., Larsson M. (2009). Odor identification deficit as a predictor of five-year global cognitive change: interactive effects with age and ApoE-*ε*4. *Behavior Genetics*.

[100] Sohrabi H. R., Bates K. A., Weinborn M. G. (2012). Olfactory discrimination predicts cognitive decline among community-dwelling older adults. *Translational Psychiatry*.

[101] Stanciu I., Larsson M., Nordin S., Adolfsson R., Nilsson L.-G., Olofsson J. K. (2014). Olfactory impairment and subjective olfactory complaints independently predict conversion to dementia: a longitudinal, population-based study. *Journal of the International Neuropsychological Society*.

[102] Devanand D. P., Michaels-Marston K. S., Liu X. (2000). Olfactory deficits in patients with mild cognitive impairment predict Alzheimer's disease at follow-up. *The American Journal of Psychiatry*.

[103] Devanand D. P., Tabert M. H., Cuasay K. (2010). Olfactory identification deficits and MCI in a multi-ethnic elderly community sample. *Neurobiology of Aging*.

[104] Bitter T., Gudziol H., Burmeister H. P., Mentzel H.-J., Guntinas-Lichius O., Gaser C. (2010). Anosmia leads to a loss of gray matter in cortical brain areas. *Chemical Senses*.

[105] Peng P., Gu H., Xiao W. (2013). A voxel-based morphometry study of anosmic patients. *British Journal of Radiology*.

[106] Bitter T., Brüderle J., Gudziol H., Burmeister H. P., Gaser C., Guntinas-Lichius O. (2010). Gray and white matter reduction in hyposmic subjects—a voxel-based morphometry study. *Brain Research*.

[107] Bitter T., Gudziol H., Burmeister H. P. (2011). Gray matter alterations in parosmia. *Neuroscience*.

[108] Gurgel R. K., Ward P. D., Schwartz S., Norton M. C., Foster N. L., Tschanz J. T. (2014). Relationship of hearing loss and dementia: a prospective, population-based study. *Otology and Neurotology*.

[109] Dawes P., Emsley R., Cruickshanks K. J. (2015). Hearing loss and cognition: the role of hearing aids, social isolation and depression. *PLoS ONE*.

[110] Mosnier I., Bebear J.-P., Marx M. (2015). Improvement of cognitive function after cochlear implantation in elderly patients. *JAMA Otolaryngology—Head and Neck Surgery*.

[111] Wong A. A., Brown R. E. (2013). Prevention of vision loss protects against age-related impairment in learning and memory performance in DBA/2J mice. *Frontiers in Aging Neuroscience*.

[112] Ono Y., Yamamoto T., Kubo K.-Y., Onozuka M. (2010). Occlusion and brain function: mastication as a prevention of cognitive dysfunction. *Journal of Oral Rehabilitation*.

[113] Rosenzweig M. R., Bennett E. L. (1996). Psychobiology of plasticity: effects of training and experience on brain and behavior. *Behavioural Brain Research*.

[114] Kerr B., Silva P. A., Walz K., Young J. I. (2010). Unconventional transcriptional response to environmental enrichment in a mouse model of Rett syndrome. *PLoS ONE*.

[115] Kondo M., Gray L. J., Pelka G. J., Christodoulou J., Tam P. P. L., Hannan A. J. (2008). Environmental enrichment ameliorates a motor coordination deficit in a mouse model of Rett syndrome—Mecp2 gene dosage effects and BDNF expression. *European Journal of Neuroscience*.

[116] Lonetti G., Angelucci A., Morando L., Boggio E. M., Giustetto M., Pizzorusso T. (2010). Early environmental enrichment moderates the behavioral and synaptic phenotype of MeCP2 null mice. *Biological Psychiatry*.

[117] Restivo L., Ferrari F., Passino E. (2005). Enriched environment promotes behavioral and morphological recovery in a mouse model for the fragile X syndrome. *Proceedings of the National Academy of Sciences of the United States of America*.

[118] Nag N., Moriuchi J. M., Peitzman C. G. K., Ward B. C., Kolodny N. H., Berger-Sweeney J. E. (2009). Environmental enrichment alters locomotor behaviour and ventricular volume in Mecp21lox mice. *Behavioural Brain Research*.

[119] Potocki L., Bi W., Treadwell-Deering D. (2007). Characterization of Potocki-Lupski syndrome (dup(17)(p11.2p11.2)) and delineation of a dosage-sensitive critical interval that can convey an autism phenotype. *American Journal of Human Genetics*.

[120] Lacaria M., Spencer C., Gu W., Paylor R., Lupski J. R. (2012). Enriched rearing improves behavioral responses of an animal model for CNV-based autistic-like traits. *Human Molecular Genetics*.

[121] McFarlane H. G., Kusek G. K., Yang M., Phoenix J. L., Bolivar V. J., Crawley J. N. (2008). Autism-like behavioral phenotypes in BTBR T + tf/J mice. *Genes, Brain and Behavior*.

[122] Moy S. S., Nadler J. J., Poe M. D. (2008). Development of a mouse test for repetitive, restricted behaviors: relevance to autism. *Behavioural Brain Research*.

[123] Moy S. S., Nadler J. J., Young N. B. (2007). Mouse behavioral tasks relevant to autism: phenotypes of 10 inbred strains. *Behavioural Brain Research*.

[124] Pearson B. L., Pobbe R. L. H., Defensor E. B. (2011). Motor and cognitive stereotypies in the BTBR T+tf/J mouse model of autism. *Genes, Brain and Behavior*.

[125] Reynolds S., Urruela M., Devine D. P. (2013). Effects of environmental enrichment on repetitive behaviors in the BTBR T+tf/J mouse model of autism. *Autism Research*.

[126] MacPherson P., McGaffigan R., Wahlsten D., Nguyen P. V. (2008). Impaired fear memory, altered object memory and modified hippocampal synaptic plasticity in split-brain mice. *Brain Research*.

[127] Yang M., Perry K., Weber M. D., Katz A. M., Crawley J. N. (2011). Social peers rescue autism-relevant sociability deficits in adolescent mice. *Autism Research*.

[128] Turner C. A., Lewis M. H. (2003). Environmental enrichment: effects on stereotyped behavior and neurotrophin levels. *Physiology and Behavior*.

[129] Schneider T., Przewłocki R. (2005). Behavioral alterations in rats prenatally exposed to valproic acid: animal model of autism. *Neuropsychopharmacology*.

[130] Schneider T., Turczak J., Przewłocki R. (2006). Environmental enrichment reverses behavioral alterations in rats prenatally exposed to valproic acid: issues for a therapeutic approach in autism. *Neuropsychopharmacology*.

[131] Baroncelli L., Braschi C., Spolidoro M., Begenisic T., Sale A., Maffei L. (2010). Nurturing brain plasticity: impact of environmental enrichment. *Cell Death and Differentiation*.

[132] Hannan A. J. (2014). Review: environmental enrichment and brain repair: harnessing the therapeutic effects of cognitive stimulation and physical activity to enhance experience-dependent plasticity. *Neuropathology and Applied Neurobiology*.

[133] Nithianantharajah J., Hannan A. J. (2006). Enriched environments, experience-dependent plasticity and disorders of the nervous system. *Nature Reviews Neuroscience*.

[134] Pang T. Y. C., Hannan A. J. (2013). Enhancement of cognitive function in models of brain disease through environmental enrichment and physical activity. *Neuropharmacology*.

[135] Ben-Sasson A., Hen L., Fluss R., Cermak S. A., Engel-Yeger B., Gal E. (2009). A meta-analysis of sensory modulation symptoms in individuals with autism spectrum disorders. *Journal of Autism and Developmental Disorders*.

[136] Hilton C. L., Harper J. D., Kueker R. H. (2010). Sensory responsiveness as a predictor of social severity in children with high functioning autism spectrum disorders. *Journal of Autism and Developmental Disorders*.

[137] Kern J. K., Trivedi M. H., Grannemann B. D. (2007). Sensory correlations in autism. *Autism*.

[138] Leekam S. R., Nieto C., Libby S. J., Wing L., Gould J. (2007). Describing the sensory abnormalities of children and adults with autism. *Journal of Autism and Developmental Disorders*.

[139] Mottron L., Dawson M., Soulières I., Hubert B., Burack J. (2006). Enhanced perceptual functioning in autism: an update, and eight principles of autistic perception. *Journal of Autism and Developmental Disorders*.

[140] Rogers S. J., Ozonoff S. (2005). Annotation: what do we know about sensory dysfunction in autism? A critical review of the empirical evidence. *Journal of Child Psychology and Psychiatry*.

[141] Tomchek S. D., Dunn W. (2007). Sensory processing in children with and without autism: a comparative study using the short sensory profile. *American Journal of Occupational Therapy*.

[142] Watling R. L., Deitz J., White O. (2001). Comparison of sensory profile scores of young children with and without autism spectrum disorders. *American Journal of Occupational Therapy*.

[143] American Psychiatric Association (2013). *Diagnostic and Statistical Manual of Mental Disorders*.

[144] Behrmann M., Avidan G., Leonard G. L. (2006). Configural processing in autism and its relationship to face processing. *Neuropsychologia*.

[145] Caron M.-J., Mottron L., Berthiaume C., Dawson M. (2006). Cognitive mechanisms, specificity and neural underpinnings of visuospatial peaks in autism. *Brain*.

[146] Gabriels R. L., Agnew J. A., Miller L. J. (2008). Is there a relationship between restricted, repetitive, stereotyped behaviors and interests and abnormal sensory response in children with autism spectrum disorders?. *Research in Autism Spectrum Disorders*.

[147] Kujala T., Lepistö T., Näätänen R. (2013). The neural basis of aberrant speech and audition in autism spectrum disorders. *Neuroscience and Biobehavioral Reviews*.

[148] Stevenson R. A., Siemann J. K., Schneider B. C. (2014). Multisensory temporal integration in autism spectrum disorders. *The Journal of Neuroscience*.

[149] Marco E. J., Hinkley L. B. N., Hill S. S., Nagarajan S. S. (2011). Sensory processing in autism: a review of neurophysiologic findings. *Pediatric Research*.

[150] Wigham S., Rodgers J., South M., McConachie H., Freeston M. (2015). The interplay between sensory processing abnormalities, intolerance of uncertainty, anxiety and restricted and repetitive behaviours in autism spectrum disorder. *Journal of Autism and Developmental Disorders*.

[151] Evans D. W., Gray F. L., Leckman J. F. (1999). The rituals, fears and phobias of young children: insights from development, psychopathology and neurobiology. *Child Psychiatry and Human Development*.

[152] Brock M. E., Freuler A., Baranek G. T., Watson L. R., Poe M. D., Sabatino A. (2012). Temperament and sensory features of children with autism. *Journal of Autism and Developmental Disorders*.

[153] Owen M. J. (2014). New approaches to psychiatric diagnostic classification. *Neuron*.

[154] Fountain C., Winter A. S., Bearman P. S. (2012). Six developmental trajectories characterize children with autism. *Pediatrics*.

[155] Bailey A., Le Couteur A., Gottesman I. (1995). Autism as a strongly genetic disorder: evidence from a British twin study. *Psychological Medicine*.

[156] Folstein S., Rutter M. (1977). Infantile autism: a genetic study of 21 twin pairs. *Journal of Child Psychology and Psychiatry and Allied Disciplines*.

[157] Ritvo E. R., Freeman B. J., Mason-Brothers A., Mo A., Ritvo A. M. (1985). Concordance for the syndrome of autism in 40 pairs of afflicted twins. *American Journal of Psychiatry*.

[158] Gaugler T., Klei L., Sanders S. J. (2014). Most genetic risk for autism resides with common variation. *Nature Genetics*.

[159] Chaste P., Klei L., Sanders S. J. (2015). A genome-wide association study of autism using the Simons Simplex Collection: does reducing phenotypic heterogeneity in autism increase genetic homogeneity?. *Biological Psychiatry*.

[160] Klei L., Sanders S. J., Murtha M. T. (2012). Common genetic variants, acting additively, are a major source of risk for autism. *Molecular Autism*.

[161] Cross-Disorder Group of the Psychiatric Genomics Consortium (2013). Identification of risk loci with shared effects on five major psychiatric disorders: a genome-wide analysis. *The Lancet*.

[162] Hultman C. M., Sandin S., Levine S. Z., Lichtenstein P., Reichenberg A. (2011). Advancing paternal age and risk of autism: new evidence from a population-based study and a meta-analysis of epidemiological studies. *Molecular Psychiatry*.

[163] Sharma R., Agarwal A., Rohra V. K., Assidi M., Abu-Elmagd M., Turki R. F. (2015). Effects of increased paternal age on sperm quality, reproductive outcome and associated epigenetic risks to offspring. *Reproductive Biology and Endocrinology*.

[164] Lundström S., Haworth C. M. A., Carlström E. (2010). Trajectories leading to autism spectrum disorders are affected by paternal age: findings from two nationally representative twin studies. *Journal of Child Psychology and Psychiatry and Allied Disciplines*.

[165] Frans E. M., Sandin S., Reichenberg A. (2013). Autism risk across generations: a population-based study of advancing grandpaternal and paternal age. *JAMA Psychiatry*.

[166] Sandin S., Hultman C. M., Kolevzon A., Gross R., MacCabe J. H., Reichenberg A. (2012). Advancing maternal age is associated with increasing risk for autism: a review and meta-analysis. *Journal of the American Academy of Child & Adolescent Psychiatry*.

[167] Marangos P., Stevense M., Niaka K. (2015). DNA damage-induced metaphase I arrest is mediated by the spindle assembly checkpoint and maternal age. *Nature Communications*.

[168] Moldrich R. X., Leanage G., She D. (2013). Inhibition of histone deacetylase in utero causes sociability deficits in postnatal mice. *Behavioural Brain Research*.

[169] Kalkman H. O. (2012). A review of the evidence for the canonical Wnt pathway in autism spectrum disorders. *Molecular Autism*.

[170] Inestrosa N. C., Varela-Nallar L. (2015). Wnt signalling in neuronal differentiation and development. *Cell and Tissue Research*.

[171] Volk H. E., Hertz-Picciotto I., Delwiche L., Lurmann F., McConnell R. (2011). Residential proximity to freeways and autism in the CHARGE study. *Environmental Health Perspectives*.

[172] Volk H. E., Lurmann F., Penfold B., Hertz-Picciotto I., McConnell R. (2013). Traffic-related air pollution, particulate matter, and autism. *JAMA Psychiatry*.

[173] Jung C.-R., Lin Y.-T., Hwang B.-F. (2013). Air pollution and newly diagnostic autism spectrum disorders: a population-based cohort study in Taiwan. *PLoS ONE*.

[174] Kalkbrenner A. E., Windham G. C., Serre M. L. (2015). Particulate matter exposure, prenatal and postnatal windows of susceptibility, and autism spectrum disorders. *Epidemiology*.

[175] Campbell D. B., Sutcliffe J. S., Ebert P. J. (2006). A genetic variant that disrupts MET transcription is associated with autism. *Proceedings of the National Academy of Sciences of the United States of America*.

[176] Volk H. E., Kerin T., Lurmann F., Hertz-Picciotto I., McConnell R., Campbell D. B. (2014). Interaction of the MET receptor tyrosine kinase gene and air pollution exposure in autism spectrum disorder. *Epidemiology*.

